# Temporal changes in genetic diversity and forage yield of perennial ryegrass in monoculture and in combination with red clover in swards

**DOI:** 10.1371/journal.pone.0206571

**Published:** 2018-11-08

**Authors:** Christophe Verwimp, Tom Ruttink, Hilde Muylle, Sabine Van Glabeke, Gerda Cnops, Paul Quataert, Olivier Honnay, Isabel Roldán-Ruiz

**Affiliations:** 1 Plant Sciences Unit, Research Institute for Agriculture, Fisheries and Food, Melle, Belgium; 2 Department of Biology, Plant Conservation and Population Biology, University of Leuven, Heverlee, Belgium; 3 Research Institute for Nature and Forest, Brussels, Belgium; 4 Department of Plant Biotechnology and Bioinformatics, Ghent University, Zwijnaarde, Belgium; Aberystwyth University, UNITED KINGDOM

## Abstract

Agricultural grasslands are often cultivated as mixtures of grasses and legumes, and an extensive body of literature is available regarding interspecific interactions, and how these relate to yield and agronomic performance. However, knowledge of the impact of intraspecific diversity on grassland functioning is scarce. We investigated these effects during a 4-year field trial established with perennial ryegrass (*Lolium perenne*) and red clover (*Trifolium pratense*). We simulated different levels of intraspecific functional diversity by sowing single cultivars or by combining cultivars with contrasting growth habits, in monospecific or bispecific settings (i.e. perennial ryegrass whether or not in combination with red clover). Replicate field plots were established for seven seed compositions. We determined yield parameters and monitored differences in genetic diversity in the ryegrass component among seed compositions, and temporal changes in the genetic composition and genetic diversity at the within plot level. The composition of cultivars of both species affected the yield and species abundance. In general, the presence of clover had a positive effect on the yield. The cultivar composition of the ryegrass component had a significant effect on the yield, both in monoculture, and in combination with clover. For the genetic analyses, we validated empirically that genotyping-by-sequencing of pooled samples (pool-GBS) is a suitable method for accurate measurement of population allele frequencies, and obtained a dataset of 22,324 SNPs with complete data. We present a method to investigate the temporal dynamics of cultivars in seed mixtures grown under field conditions, and show how cultivar abundances vary during subsequent years. We screened the SNP panel for outlier loci, putatively under selection during the cultivation period, but none were detected.

## Introduction

Whereas it is widely accepted that plant species richness and plant trait diversity have a positive effect on the functioning of ecosystems, recent progress in community ecology has also emphasized the importance of intraspecific diversity [1–4]. Positive relationships between ecosystem functioning and diversity at both the inter- and intraspecific level are known as *diversity effects*. Such diversity effects result from functional complementarity among members of the same or different species, resulting in structural, trophic or phenological niche differentiation. A large part of the Earth’s terrestrial ecosystem is covered with grasslands [5], which support a wide range of ecosystem services including forage production, water regulation, maintenance of soil fertility and structure, carbon sequestration, and provisioning of habitat to many plant and animal species [6]. These services strongly depend on the grasslands’ ecosystem functioning, which in turn is affected by the diversity they harbor, i.e. by the variety of species, functional traits and genes present in the plant community [7]. Identification of relevant diversity effects in grassland communities and understanding the underlying mechanisms may support effective management strategies and sustainable grassland exploitation.

Grasslands can develop progressively upon grazing or mowing activity, but in highly productive livestock systems they are sown for the production of high quality forage. These agronomic grasslands support the production of meat and dairy products, and the total value of grass production in the EU is estimated at more than 23 billion Euro [8]. Such grasslands in Europe are often dominated by species of the genus *Lolium* (accounting for about 23% of the grasslands), with *L*. *perenne* (perennial ryegrass) as the most prevalent species [9]. Cultivation of perennial ryegrass may comprise sowing one or a mixture of cultivars, combinations with other grass species such as timothy (*Phleum pratense*) or tall fescue (*Festuca arundinacea*), or with legumes such as white clover (*Trifolium repens*) or red clover (*Trifolium pratense*). Although perennial ryegrass is still frequently cultivated as a monoculture, sustainable agricultural practice is progressing towards combinations of ryegrass with legumes, which reduces the need for nitrogen fertilizer application [10, 11]. The close interaction between intermingled grass and legume plants has a synergistic effect on biomass production, and mixed species swards can deliver higher yields than would be predicted from their component monocultures [12]. While monospecific swards can produce high quality forage under favorable environmental and soil fertility conditions, multispecies swards may increase resistance and resilience against environmental disturbances such as persistent periods of drought [13, 14]. Especially in a context of global climate change, where such disturbances are expected to become more frequent [15], increasing diversity might prove to be key for the sustainable production of high quality forage.

The importance of intraspecific diversity for the functioning of cultivated grasslands has not been investigated in depth so far. However, the intraspecific diversity of perennial ryegrass might be an important factor for sward productivity and resilience, as cultivars of this species are typically genetically very diverse [16–18]. Ryegrass cultivars are commonly derived from multiple parental components due to the necessity to incorporate a sufficiently high number of self-incompatibility alleles (at the self-incompatibility loci S and Z), to allow abundant seed set [9, 19–21]. Furthermore, genetic diversity benefits performance [22]. Given this high level of genetic diversity of the sown individuals, the genetic composition of the grassland sward is likely to change during the cultivation period. Changes might be driven by self-thinning or density-dependent mortality [23], or by the selection of specific genotypes that are better adapted to the prevailing conditions (for example, differences in early vigor, tolerance to defoliation, or competitive ability). Therefore, understanding temporal changes in the genetic composition and diversity is essential to get a more complete understanding of the complex plant-to-plant interactions in highly productive grassland swards. Furthermore, understanding changes in the genetic composition of highly productive ryegrass grasslands in response to particular management practices can be of high significance to breeding applications in at least two ways. First, by providing information on the proportion of genetic diversity originally present in the seed composition that remains present in the field. Currently, the necessity to incorporate genetic diversity in ryegrass cultivars compromises to a certain extent the selection intensity that can be applied. Knowing what proportion of this diversity remains present in the ryegrass population after establishment could guide the optimization of breeding programs by allowing a more precise fine-tuning of the balance between genetic diversity and selection intensity. Second, revealing the identity of particular genes, and corresponding alleles, that are preferentially selected under specific circumstances could enable breeders to target traits associated with performance and to exploit polymorphisms in these genes during selection.

Here, we monitored changes in the genetic diversity and forage yield of cultivated ryegrass populations over the course of four years. Experimental field plots were established with populations of perennial ryegrass, with or without red clover. We simulated different levels of potential niche differentiation by mixing phenotypically contrasting cultivars in different combinations, i.e. low vs. high tillering cultivars for the ryegrass component [24], and erect vs. creeping growth habit cultivars for the clover component [25]. We used genotyping-by-sequencing (GBS) of pooled samples (pool-GBS) to quantify genome-wide allele frequencies. This approach has recently been applied to perennial ryegrass to differentiate cultivars [26] and to characterize the genetic basis of flowering time and crown rust resistance [27]. Because we here specifically aimed to compare measurements of genetic diversity among single populations at different time points, we empirically validated this method with special emphasis on SNP data completeness, allele frequency accuracy and removal of non-reproducible SNPs. Our specific objectives were: (1) Investigating whether the composition of ryegrass and clover cultivars of the initial seed mixtures affects forage production and abundance of the clover and ryegrass components. (2) Validating the reliability of GBS to characterize the genetic diversity of the ryegrass component of grassland swards using pooled samples. We compared alternative allele frequencies (AAF) estimated from pooled samples (AAF_pool_) with AAF calculated from the constituent individual samples (AAF_ind_). (3) Characterizing the temporal changes in genetic diversity of the ryegrass component during the cultivation period, based on AAF_pool_. (4) Screening for the presence of outlier SNP loci, which may be indicative for selection of specific alleles during cultivation. (5) Investigating how temporal changes in genetic diversity of the perennial ryegrass component relate to the composition of the initial seed mixture, forage production and abundance of the ryegrass and clover components.

## Material and methods

### Field trial

A field trial was established in April 2011 on a sandy loam soil in Merelbeke, Belgium (50.9867 N 3.7912 E). Seven different seed compositions (Table 1) were sown in plots of 6 by 1.8 m according to a randomized complete block design with two replicates, rendering 14 field plots. Not all possible combinations of cultivars were included in the seed compositions. Seed compositions 1 and 2 were monospecific and consisted of the ryegrass cultivars Merks and Meloni, respectively. Merks is high tillering, late heading, and was derived from a polycross with three components. Meloni is low tillering, intermediate heading and was derived from a pair cross. Seed compositions 3 to 7 comprised both perennial ryegrass and red clover. Two red clover cultivars were used. Crossway was chosen because of its creeping growth habit; Lemmon was chosen because of its erect growth habit. Compositions 3 and 4 consisted of both red clover cultivars combined with either Merks or Meloni. Compositions 5, 6, and 7 consisted of both ryegrass cultivars with either Lemmon plus Crossway, Lemmon, or Crossway, respectively. Sowing densities were 1400 seeds/m^2^ for the ryegrass monoculture plots and 1190 seeds/m^2^ for the mixed species plots, with a ratio of 70 perennial ryegrass seeds to 30 red clover seeds, as is common agricultural practice.

**Table 1:** Seed compositions.

Species		Perennial ryegrass		Red clover	
Cultivar		Merks	Meloni	Lemmon	Crossway
n parental components		3	2	-	-
Growing habit		high tillering	low tillering	erect	creeping
Heading		late	intermediate	-	-
Seed composition (%)	1	100	-	-	-
	2	-	100	-	-
	3	70	-	15	15
	4	-	70	15	15
	5	35	35	15	15
	6	35	35	30	-
	7	35	35	-	30

Seven seed compositions were sown in replicate, rendering 14 field plots in total. Seed compositions 1 and 2 are ryegrass monocultures of Merks and Meloni respectively. By mixing the two ryegrass cultivars together with red clover in a 70/30 seed ratio, we created five multi-species mixtures with different combinations of cultivars. The two red clover cultivars are Lemmon and Crossway. A single batch of each cultivar was used for sowing.

The trial was fertilized and weeded according to common agricultural practice (S1 Table), and mown with a Haldrup plot harvester (Haldrup GmbH, Ilshofen GER). Three cuts were harvested in 2011 (year 1), four cuts in 2012 (year 2) and five cuts in 2013 (year 3) and 2014 (year 4). The total harvest of each cut was weighted to determine the fresh weight. A subsample of approximately 450 g was dried in a ventilated oven at 65°C during 48 h to determine its water content. This value was subsequently used to estimate the herbage dry matter weight (DMW; t/ha). The botanical composition of mixed species swards was determined right before each cut by collecting four subsamples of approximately 350 g per plot to account for local heterogeneity. The subsamples were separated manually into three fractions: grass, clover and weed (a minor weed fraction was harvested in the first year, but was negligible in subsequent years). Each fraction was dried in a ventilated oven at 65°C during 48h, and the respective dry weights were determined. The portion of each fraction was averaged over the four subsamples.

**Fig 1:**
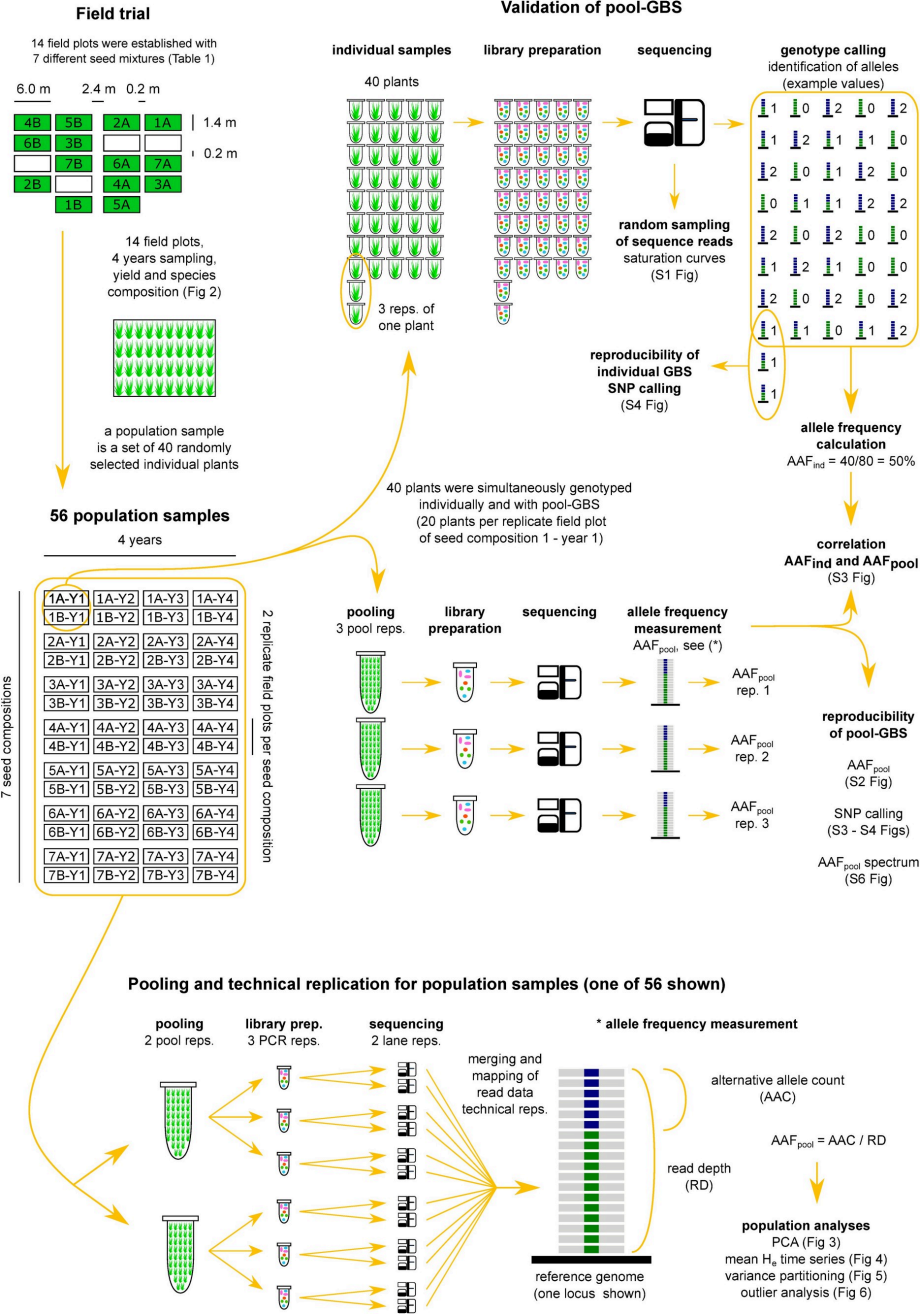
Schematic illustration of the experimental setup of this study. An overview of the field trial, the perennial ryegrass population samples and the pooling and replication strategy is provided. For GBS of individual plants, genotypes are called per plant, i.e. homozygote reference (0), heterozygote (1) or homozygote alternative (2). The alternative allele frequency (AAF_ind_) in the set of 40 individual plants is the sum of alternative alleles, divided by the total number of chromosomes investigated (80 in this case). For pool-GBS, the alternative allele frequency (AAF_pool_) is directly measured from the read data, i.e. the alternative allele read count divided by the total read depth (RD) of a locus. For genotyping the 56 population samples, leaf tissue was weighed and pooled in replicate. DNA was extracted from each pooled sample (112 in total), and libraries were prepared. The amplification step was done in three separate PCR reactions, and each PCR product was split and sequenced on two separate HiSeq lanes. Thereafter, sequence read data was merged for each of the original 56 population samples, and AAF_pool_ was measured for population genetic analysis.

Leaf samples for genetic analysis were collected in 2011 after establishment of the 14 plots, and immediately before the spring cut during three subsequent years (2012–2014). At each sampling moment, 40 perennial ryegrass leaves were collected in each plot, rendering a total of 56 sets of 40 leaves (Fig 1). Leaves were picked randomly, but sampling the same plant twice was avoided by maintaining a minimum distance of at least 15 cm between sampling positions. Individual leaf samples were immediately frozen at -80°C, freeze-dried and vacuum packed for storage. With this sampling strategy, we intended to get a representation of the genotypes present in the sward at a given moment in time.

### Genotyping-by-sequencing of pooled samples (pool-GBS)

#### Pooling strategy

The pool-GBS procedure was validated on a set of 40 plants derived from seed composition 1 (20 plants of replicate A and 20 plants of replicate B, both sampled in 2011). The 40 individual leaf samples were genotyped individually and allele frequencies were calculated (Fig 1) as described below. This information was compared to allele frequencies derived from three replicate pools, each containing equal weights (5 mg) of leaf tissue of the same 40 plants (Fig 1). For one individual sample, three GBS libraries were generated to estimate the reproducibility of the GBS procedure on an individual genotype basis. Comparison of the three replicated pools allowed us to estimate the reproducibility of pool-GBS. AAF derived from individual samples, from the pools, and from pairwise merging of replicate pools were compared to assess the accuracy of allele frequency estimation and reproducibility of SNP calling of the pool-GBS procedure. Based on the results of this validation step (see Results section), we developed the following pooling strategy for the samples of the field experiment: two replicate tissue pools were created for each of the 56 sets of 40 leaves, by weighing 5 mg from each leaf sample of the respective plots, yielding a total of 112 pooled samples.

#### GBS library preparation

DNA of the 40 individuals and three pools of the validation set was isolated with the Bio-Nobile Quickpick Plant DNA extraction kit. DNA of the 112 tissue pooled samples was isolated using the CTAB procedure of Doyle [28]. DNA integrity was checked by gel electrophoresis and the concentration was measured with Quantifluor intercalating dye on a Promega Quantus fluorometer (Promega, Madison, USA). All samples were genotyped using a single-enzyme GBS procedure based on Elshire [29] and Byrne [26]. In short, 100 ng of genomic DNA was digested with *Pst*I (New England Biolabs, Ipswitch, USA), and barcoded adapters were ligated with T4 ligase (New England Biolabs, Ipswitch, USA) in a final volume of 50 μL. Ligation products were purified with AM-pure magnetic beads [30] and eluted in 50 μL TE. PCR amplification was performed separately for each adapter-ligated sample. For the validation experiment we used an aliquot of 2 μL of the bead-purified ligate as template for PCR amplification. The fragment size distribution of amplified libraries was evaluated using a Qiagen QIAxcel system (Qiagen, Venlo, NL). Libraries were quantified with the Quantus fluorometer, and then normalized, pooled, bead-purified, and 100 bp paired-end sequenced on an Illumina Hiseq2000 instrument by BGI (Beijing, CN). Because of the sequencing of short fragments, only the forward reads were used for the data analysis.

The protocol for the 112 tissue pool samples was slightly adjusted, based on the results of the validation step. In this case, the 50 μL bead-purified ligate was split into three aliquots, and 16.7 μL was used for three replicate PCR reactions to account for PCR amplification bias. Amplified libraries were quantified as described above and pooled in equal amounts into three ‘super’ libraries (one for each set of replicated PCR reactions). Super libraries were again bead-purified and 100 bp single-end sequenced on two parallel lanes per super library on a Hiseq2500 instrument (Genomic Services Lab at HudsonAlpha, Huntsville, USA) (Fig 1). In this case we used single-ended sequencing because the results of the validation experiment indicated that very short insert sizes were sequenced (+/- 100 bp). This means that a large proportion of read pairs overlapped at the 3’ side, resulting in read-through (sequencing of adaptor sequences). Moreover, the overlapping part of read pairs was redundant as they were observations of the same molecule (sequenced twice).

#### NGS read data processing, mapping and analysis

Reads were demultiplexed with GBSX 1.3 [31] allowing 1 mismatch in the barcodes. Sequence quality was checked with FastQC [32] and summarized with MultiQC v0.7 [33]. Reads containing uncalled bases (Ns) were discarded using a custom python script. Reads with average base quality below 35 were discarded with prinseq-lite 0.20.4 [34]. 3’ restriction site remnants and common adapter sequences were removed with Cutadapt [35]; 5’ restriction site remnants were removed with FASTX-Toolkit 0.0.13 [36]. All reads were trimmed to a maximum length of 86 bp to account for variable barcode lengths. Reads shorter than 50 bp after trimming were discarded. Trimmed reads were aligned to the perennial ryegrass reference genome [19] with the BWA-mem algorithm in BWA 0.7.8 with default parameters [37]. Alignments were sorted, indexed, and filtered on mapping quality 20 with SAMtools 1.2. [38]. For each of the 56 samples, the 12 BAM files were merged (i.e. two tissue pool replicates x three PCR replicates x two sequencing lane replicates), yielding 56 BAM files for further analysis. These BAM files were converted to mpileup format with SAMtools, while filtering on minimum RD 30. All previous steps were parallelized with GNU parallel [39].

For each genomic position, we counted the number of missing data across samples. For the individual plants, we filtered on maximum of 1, 5 or 10 missing genotype calls per locus. For the pool samples, we filtered the loci on > 0 missing data. The three datasets, individual plants, pool replicates of the validation and 56 pools of the field experiment were also filtered on excessive RD (e.g. positions with extremely high number of reads, which probably represent repetitive regions, were removed). The thresholds for this filtering step were determined for each dataset separately; maximum 6 k total RD for the dataset of 40 individual samples (and replicate individual samples), 3.5 k for the three replicate pools of the validation experiment, 7 k for the pairwise merged pools and 150 k for the 56 pooled samples of the field experiment.

RD saturation curves were constructed by first merging all read data of the validation experiment, followed by computational subsampling of reads, read mapping, and calculating the number of genomic reference positions with a minimal RD of 10, 30, 100, or 300 reads at increasing numbers of reads mapped.

#### SNP calling and allele frequency measurement

For individual plants, SNPs were called with GATK HaplotypeCaller 3.3.2 [40]. Indels and multi-allelic SNPs were removed and genotype calls below RD10 or genotype quality (GQ) score below 30 were flagged as missing data with VCFtools 0.1.14 [41]. AAF_ind_ at each SNP position over the set of 40 individual plants was calculated as the number of genotypes with the alternative allele, counting homozygous reference calls as 0, heterozygous calls as 1, homozygous alternative calls as 2. This value was divided by the number of sampled chromosomes. To identify SNPs and estimate AAF_pool_ of the pooled samples, we used SNAPE-pooled. This is a Bayesian method to differentiate genotyping errors from real alleles on a per-sample basis, using the Watterson’s estimator theta as a diversity prior determined by NPstat [42, 43]. The theta value was calculated for each pooled sample separately, with a fixed minimum minor allele count (MAC) of three reads. SNAPE-pooled was run with an informative prior and folded spectrum. The algorithm estimates for each sample the AAF_pool_ values based on allelic RDs, and assigns probabilities to an allele being fixed reference if 1− p(0) < 0.9 or fixed alternative if p(1) > 0.9. A custom python script was used to merge SNAPE-pooled output files to a single AAF_pool_ matrix and set frequencies to zero if 1− p(0) < 0.9 and to one if p(1) > 0.9, where p represents the posterior probability as described in Raineri [42]. After all the above filters had been applied, we removed positions that were tri- or tetra-allelic across all samples.

For the validation experiment, we calculated the Pearson’s correlation and the median deviation between AAF_ind_ and AAF_pool_ for SNPs that were identified by both approaches.

### Data analysis

#### Herbage DMW and species composition

**Fig 2:**
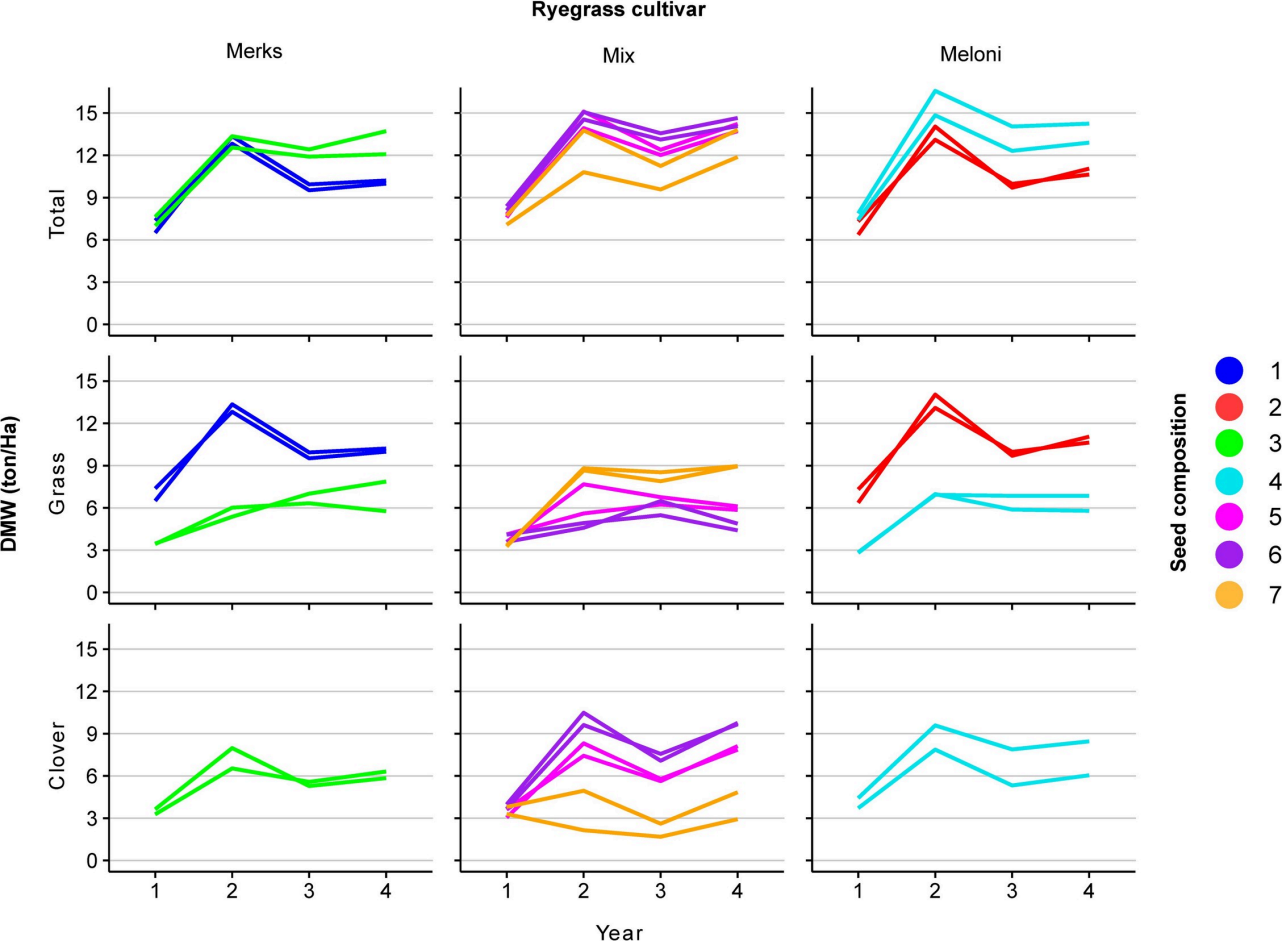
Forage yield harvested per year. The first row shows the DMW of the total yield (both species), the second and third row show the DMW of respectively the grass component and the clover DMW. The first column shows the seed compositions containing the ryegrass cultivar Merks, the second column shows the seed compositions containing both ryegrass cultivars, and the third column shows the seed compositions containing the ryegrass cultivar Meloni.

The herbage DMW harvested each year (Fig 2) was analyzed with linear mixed models implemented in R 3.4.0 [44] with the *lmer* function of the *lme4* package (version 1.1–13) [45]. To test the effects of species or cultivar on the total DMW three hypotheses were formulated (Table 2). Seed compositions 1 to 4 allow testing the contrast between the ryegrass cultivars and the presence or absence of red clover (H1). Seed compositions 3 to 5 allow to test for the effect of the ryegrass component when combined with both red clover cultivars (H2). Finally, seed compositions 5 to 7 allow to test the effect of the clover component when combined with both ryegrass cultivars (H3). The significance of the variables expressing the hypotheses were determined with likelihood ratio tests implemented with the *anova* function. The following three models were tested:

$$Y_{lmjk}=\mu +L_{l}+T_{m}+(L_{l}\times T_{m})+R_{j}+Y_{k}+\epsilon_{lmjk}\;(model\;1,H1)$$

$$Y_{ljk}=\mu +L_{l}+R_{j}+Y_{k}+\epsilon_{ljk}\;(model\;2,H2)$$

$$Y_{mjk}=\mu +T_{m}+R_{j}+Y_{k}+\epsilon_{mjk}\;(model\;3,H3)$$

**Table 2:** Effects of the perennial ryegrass and red clover cultivars on forage yield and species abundance.

Hypothesis		Model	DMW	Seed comp.	Component	Chi-square	d.f.	pLRT
H1	a	1	total	1–4	L_l_ x T_m_	2.12	1	0.145
	b	1	total	1–4	L_l_	5.37	1	0.021
	c	1	total	1–4	T_m_	22.39	1	2.23 x 10^−6^
H2	a	2	total	3–5	L_l_	8.33	2	0.015
	b	2	grass	3–5	L_l_	0.14	2	0.934
	c	2	clover	3–5	L_l_	6.98	2	0.031
H3	a	3	total	5–7	T_m_	17.56	2	1.5 x 10^−4^
	b	3	grass	5–7	T_m_	16.83	2	2.2 x 10^−4^
	c	3	clover	5–7	T_m_	24.71	2	4.3 x 10^−6^

Three hypotheses were formulated (see Material and Methods). H1 tests the effects of the ryegrass cultivar and the presence of red clover in field plots containing a single ryegrass cultivar (H1), the effect of the ryegrass component in field plots containing a mixture of two red clover cultivars (H2), and the effect of the clover component in field plots containing a mixture of two ryegrass cultivars (H3). Each hypothesis is subdivided in three tests that use the same model. DMW indicates whether the yield of both species (‘total’), or the ryegrass and clover fractions were used as dependent variable. Seed composition indicates which field plots were considered for each test. Component is the predictor variable that was tested with the likelihood ratio test. Chi-square, d.f. and pLRT are respectively the test statistic, degrees of freedom and probability of the likelihood ratio test.

In model 1, Y_lmjk_ represents the total DMW (both species) of field plot replicate *j* of the seed composition with ryegrass cultivar *l* (Merks or Meloni) and clover component *m* (a mixture of the two red clover cultivars, or no clover) in year *k* (1 to 4). (L_l_ x T_m_) is the interaction between L_l_ and T_m_, which are both fixed effects. R_j_ (replicate) and Y_k_ (year) are random effects. The term μ represents the mean total DMW, and ϵ_ij_ is the error term. Model 2 is similar to model 1, but the clover component (and the interaction term) have been excluded. In this case, the ryegrass component *l* is either Merks, Meloni or a mixture of both cultivars. Model 3 is similar to model 2, but in this case L_l_ has been replaced by T_m_. In this case, the clover component *m* is either Lemmon, Crossway or a mixture of both cultivars. Additionally, H2 and H3 were tested considering the DMW of grass and clover separately.

#### Genetic composition and diversity of the ryegrass component

The SNPs were filtered on minimum 10% and maximum 90% mean AAF_pool_ per locus across the 56 samples (see Results section for justification). Principal component analysis (PCA) was then used to detect the most pronounced trends in the data. We used the *prcomp* function (3.0.2) on the centered, non-scaled AAF_pool_ data (samples were oriented as rows and SNPs as columns). Hence, the scores on the principal components represent the 56 samples, i.e. 14 field plots at 4 time points, and a lineplot of year against score for one of the principal components represents shows changes in the genetic composition of each field plot over time.

The expected heterozygosity was calculated per locus and sample, based on the AAF_pool_ values following *H_e_* = 2 × *AAF_pool_* × (1 − *AAF_pool_*). Subsequently, the genetic diversity contained in each sample was calculated by averaging H_e_ over all SNPs (i.e. mean H_e_). As each plot was sampled at four discrete time points, the data were organized in 14 temporal series (corresponding to the 14 field plots) for further inspection. H_e_ values are independent of the alternative allele being the minor or major allele, and allow comparing genetic diversity in samples corresponding to different field plots, or in samples corresponding to different time points of the same field plot. Mean H_e_ is maximal (0.50) when all alleles occur in equal proportions and decreases as the frequency of one of the allele (either reference of alternative) increases. A low mean H_e_ value might thus be indicative of an overall lower level of diversity when samples of different plots are compared. Correspondingly, changes in mean H_e_ might be indicative of selection when samples were compared that were taken at different time points. Mean H_e_ data were analyzed with a linear model with the *aov* function in R (model 4).

$$mean\;H_{e\;ijk}=\mu +S_{i}+(S_{i}\times R_{j})+(S_{i}\times Y_{k})+\epsilon_{ijk}\;(model\;4)$$

With mean H_e ijk_ representing the genetic diversity of field plot replicate *j* (A or B) of seed composition *i* (7 in total) in year *k* (1 to 4). The factor S_i_ represents seed composition, and the interaction of seed composition and replicate (S_i_ x R_j_) represents differences of AAF_pool_ between replicate field plots (irrespective of time). The interaction of seed composition and year (S_i_ x Y_k_) captures temporal changes in mean H_e_, which are consistent across replicate field plots of a given seed composition. Plot-specific changes of mean H_e_ are captured by the residual term ϵ_ijk_. The degrees of freedom of this model are 55 for the dependent variable mean H_e_, 6 for S_i_, 7 for (S_i_ x R_j_), 21 for (S_i_ x Y_k_) and 21 for the residuals.

Next, we investigated possible temporal changes in the relative abundance of the two ryegrass cultivars in the plots in which both were sown together (seed compositions 5 to 7), using cultivar private SNPs. We considered ‘Merks private SNPs’ those that are not polymorphic in plots in which only Meloni was sown, but polymorphic in plots where Merks was sown (either as a single ryegrass cultivar or in mixture with Meloni). Conversely, Meloni private SNPs were not polymorphic in plots in which only Merks was sown, but polymorphic in plots where Meloni was sown. The mean AAF_pool_ per sample was calculated for both private SNP sets and represented graphically for inspection.

#### Temporal changes of allele frequencies

The time series of AAF_pool_ frequencies were analyzed with a similar approach as for mean H_e_ (described above). H_e_ reflects the balance in allele frequency of two alleles at a single locus. This on its own does not inform us about the identity of the allele that is more or less abundant. Linear regression of AAF_pool_ values allows investigating these aspects. The AAF_pool_ data was analyzed with the *aov* function using the following model:

$${AAF}_{pool\;ijk}=\mu +PC1+S_{i}+(S_{i}\times R_{j})+(S_{i}\times Y_{k})+\epsilon_{ijk}\;(model\;5)$$

Model 5 is similar as model 4, except for an additional covariate PC1 that represents the scores of the first principal component of the PCA. In this model, PC1 captures the differentiation between Merks and Meloni, and changes in the relative abundance of these two cultivars in the plots in which they were sown together (seed compositions 5 to 7). This was considered necessary after inspection of the PCA results and the temporal changes of mean H_e_ (see Discussion for further explanation). The variance of AAF_pool_ was partitioned to estimate the relative importance of the interaction between seed composition and year (S_i_ x Y_k_). This component captures the variance attributed to temporal changes in AAF_pool_ that are consistent for field plots of the same seed composition.

#### Identification of outlier loci

Finally, we screened the SNPs for outliers, i.e. loci that were putatively under selection during the cultivation period. The allele frequencies of loci under selection (or linked with unobserved loci under selection), are expected to change more dramatically over time than those of neutral loci. The quotient of the mean sum of squares (MSS) of (S_i_ x Y_k_) and the residual sum of squares (RSS) (model 5) was used as test statistic. This value represents the ratio of variance explained by changes in allele frequency that are consistent for replicated field plots of the same seed composition to changes that are not consistent. This model assumes that the ryegrass component of replicate field plots experiences similar selection pressures and responds similarly. We compared several probability density functions to fit the null distribution of the test statistic, including the chi-square, F, gamma and log-normal distribution (see Results). The *fitdistr* function of the *MASS* package of R (version 7.3–47) was used for maximum likelihood-fitting of the distributions (Venables and Ripley, 2002).

## Results

### Herbage yield and species abundance

The total herbage DMW showed a similar temporal pattern across field plots (Fig 2). In general, the establishment year was the least productive, while the second year was the most productive.

Hypothesis 1 was tested using seed compositions 1 to 4. Both the ryegrass cultivar (either Merks or Meloni) and the presence of red clover significantly affected the herbage yield (Table 2, H1; pLRT = 0.021 and pLRT = 2.23 x 10^−6^ respectively). The interaction of these effects was not significant (pLRT = 0.145). The total DMW was higher when perennial ryegrass was sown in combination with red clover (Fig 2; seed compositions 3 > 1 and 4 > 2). The DMW of the Meloni monoculture was higher than that of Merks, and this was more evident in the plots in which Merks or Meloni were combined with red clover (seed compositions 4 > 3).

Significant effects of the ryegrass component were detected for DMW (Table 2, H2a, pLRT = 0.015), when sown in combination with a mixture of both red clover cultivars. When the effect of the ryegrass component on the DMW of grass and clover was tested separately, a significant marginal effect was only detected for the clover DMW (pLRT = 0.031).

For field plots in which a mixture of both ryegrass cultivars was sown in combination with red clover (seed compositions 5 to 7), plots containing only Lemmon had the highest yield, followed by mixed red clover cultivars, and finally Crossway (seed composition 6 > 5 > 7). The effect of red clover composition was significant for the total DMW, and for the grass DMW and clover DMW separately (Table 2, H3, pLRT = 1.5 x 10^−6^; pLRT = 2.2 x 10^−3^; pLRT = 4.3 x 10^−6^ respectively). However, the effect on the DMW of both species separately was reversed. The highest yield of clover was obtained with Lemmon, and the lowest yield of ryegrass was obtained with Crossway (Fig 2).

Taken together, these results indicate that the presence of red clover had a significant effect on the total DMW. Meloni was higher yielding than Merks in ryegrass monocultures and in combination with red clover. In combinations with perennial ryegrass, Lemmon (erect growing habit) displayed a better competitive ability than Crossway (creeping growing habit), resulting in a larger total DMW although the yield of the grass component was significantly lower when combined with Lemmon.

### Comparison of pool-GBS allele frequencies and frequencies estimated from GBS genotyping of individuals

First, the pool-GBS procedure was validated using a subset of 40 leaf tissue samples. GBS sequencing resulted in 2.4 ± 0.4 M reads for the individual plants (n = 40) and 14.1 ± 1.1 M reads for the pools used for validation (n = 3). To determine the optimum number of reads per sample, we constructed RD saturation curves at varying levels of minimum RD threshold. The saturation curves suggest that the majority of potentially available GBS loci are covered if at least ~20 M reads are obtained per sample and that the coverage increases when data of replicate pools are merged (S1 Fig).

Comparison of the genotype calls of three technical replicates of a single plant (replicate GBS library preparation and sequencing) shows that individual genotyping was highly reproducible. On average 93.6% of the genotype calls were identical in the three pairwise comparisons and 92.4% of the genotype calls was identical across all three replicate SNP sets (S3E Fig). Next, we compared the alternative allele frequencies based on the genotyping data of 40 individual plants (AAF_ind_) to the allele frequencies estimated in three replicate pools (AAF_pool_). Because the 40 individual plants were not sequenced to saturation (S1A Fig), we considered three thresholds of missing data across the individuals, i.e. max. 1, 5 or 10 missing plants (out of 40) per SNP (S2A Fig, columns), and compared them to replicate pool 1 at increasing minimum RD threshold per SNP position in the pool data, i.e. minimum 30, 100 and 300 reads per SNP position (S2A Fig, rows). In general, correlations were high, with r ranging from 0.94 to 0.96. As the maximum missing data threshold for the AAF_ind_ becomes less stringent and more SNPs are considered in the correlation, r slightly decreased. As the minimum RD threshold for the pool increased, less SNPs are considered and r slightly increased (S2A Fig). Therefore, the allele frequencies measured in one pool agreed very well with those estimated by individual genotyping. This was consistent for the three pool-GBS replicates (S2B Fig), and shows that maximizing the number of loci screened by using a RD threshold of 30 for SNAPE-pooled does not negatively affect the AAF_pool_ accuracy. Next, we analyzed the correlation of the AAF_pool_ of two pool-GBS replicates, considering only the SNP positions that were also identified in the individuals. All three pairwise AAF_pool_ comparisons showed a slightly lower r than AAF_ind_ versus AAF_pool_ comparisons (S2C Fig). The highest correlation (r > 0.967) with the AAF_ind_ was achieved when read data from two replicate pools were merged before SNP calling (S2D Fig). Therefore, we decided to create two replicates of leaf tissue pools for the genotyping of the 56 population samples.

### Reproducibility of SNP identification

More than half of the SNPs were called uniquely by SNAPE-pooled in a given pool sample but were not identified by GATK in the set of 40 individuals even though sufficient RD was available on the respective loci (S3A Fig). This was consistent across the three pool replicates (S3B Fig). Likewise pairwise comparisons of replicate pools showed that up to 29% of the SNPs was uniquely identified by SNAPE-pooled in a given pool, but not in a replicate pool sample, despite weighing material from the same 40 leaves (S3C Fig). Combining the read data of two pool replicates showed similar patterns (S3 Fig, compare B and D). Furthermore, intersecting the SNAPE-pooled SNP sets of the three pool replicates showed that 59.8% of all SNPs were called uniquely in a single pool, and only 31.9% were common to the three pools (S3E Fig).

We ran the NPstat/SNAPE-pooled pipeline with a range of parameter values to test if the number of non-reproducible SNPs could be reduced (results not shown). The minor allele read count (MAC) threshold of NPstat was increased from minimum 2 (default) up to minimum 6, which resulted in (reproducible) estimates for theta from ca. 0.014 to ca 0.007. However, varying the theta diversity prior for SNAPE-pooled in this range did not reduce the number of non-reproducible SNPs in the output of SNAPE-pooled.

In order to identify the source of the non-reproducible SNP calls, we compared the AAF spectra of uniquely called (non-reproducible) SNPs versus SNPs that were consistently called (reproducible) by SNAPE-pooled in replicate pools. Pairwise comparisons of replicate pools revealed that non-reproducible SNPs were strongly skewed towards low AAF_pool_ values (S4 Fig). For instance, 5.1% to 5.8% of the reproducible SNPs and 65.6% to 69.7% of the non-reproducible SNPs had an AAF_pool_ < 3% across the various pairwise pool comparisons. Taken together, these data suggest that non-reproducible SNPs are derived from randomly distributed and typically low-frequency read errors.

In conclusion, SNP filtering based on p(0) and p(1) values as recommended by SNAPE-pooled needs to be complemented with additional filtering based on the allele frequency spectrum. We chose a cutoff of minimum 10% < mean AAF_pool_ < 90% for at least one sample out of 56 as criterion to retain SNPs for further data analysis.

### SNP genotyping of the 56 pooled samples

Based on the insights obtained in the validation experiment, we used the strategy of creating two replicate pools of tissue, preparing one GBS library per tissue pool, performing three independent PCRs per library, sequencing all of those in parallel on two lanes, and merging the read data per pooled sample to estimate AAF_pool_ profiles of the 56 samples of the field experiment (Fig 1).

Sequencing resulted in 29.3 ± 5.2 M reads per pooled sample, which cover on average 2.3 ± 0.2 MB of the reference genome with sufficient RD to estimate AAF_pool_ (RD > 30) (Raineri [42], and results of the validation experiment) (S1B Fig). In total, 3.76 Mbp of the reference genome sequence was covered by reads from at least one sample. However, we expected that comparing genetic diversity of samples is more accurate when a common set of loci is considered. Therefore, we only considered loci with complete data (56 AAF_pool_ values), which cover ca. 1.1 Mb of the reference genome sequence. We removed approximately 15 kb of the base positions were covered with excessive total RD (above 150 k reads over all samples), and represent genomic repeat sequences. The remaining read data covered ca. 1.085 kb, which represents ca. 0.1% of the 1.13 Gbp reference genome sequence [19].

SNAPE-pooled identified 238 k SNPs. We removed 9 k SNPs with more than two alleles across all samples. As expected from the validation experiment, many SNPs which were polymorphic in just one sample had a low AAF_pool_. To reduce the fraction of non-reproducible SNPs (see validation), we only retained SNPs with a mean AAF_pool_ per SNP of minimum 10% and maximum 90%, resulting in a dataset of 22,324 SNPs. Usually, heteroscedastic allele frequencies are normalized [46]. However, we preferred to work with non-normalized allele frequencies because normalization inflates AAF_pool_ values close to either zero or one and/or present in only a few samples, which are abundant in our dataset.

### Overall patterns of differentiation among populations based on PCA

**Fig 3:**
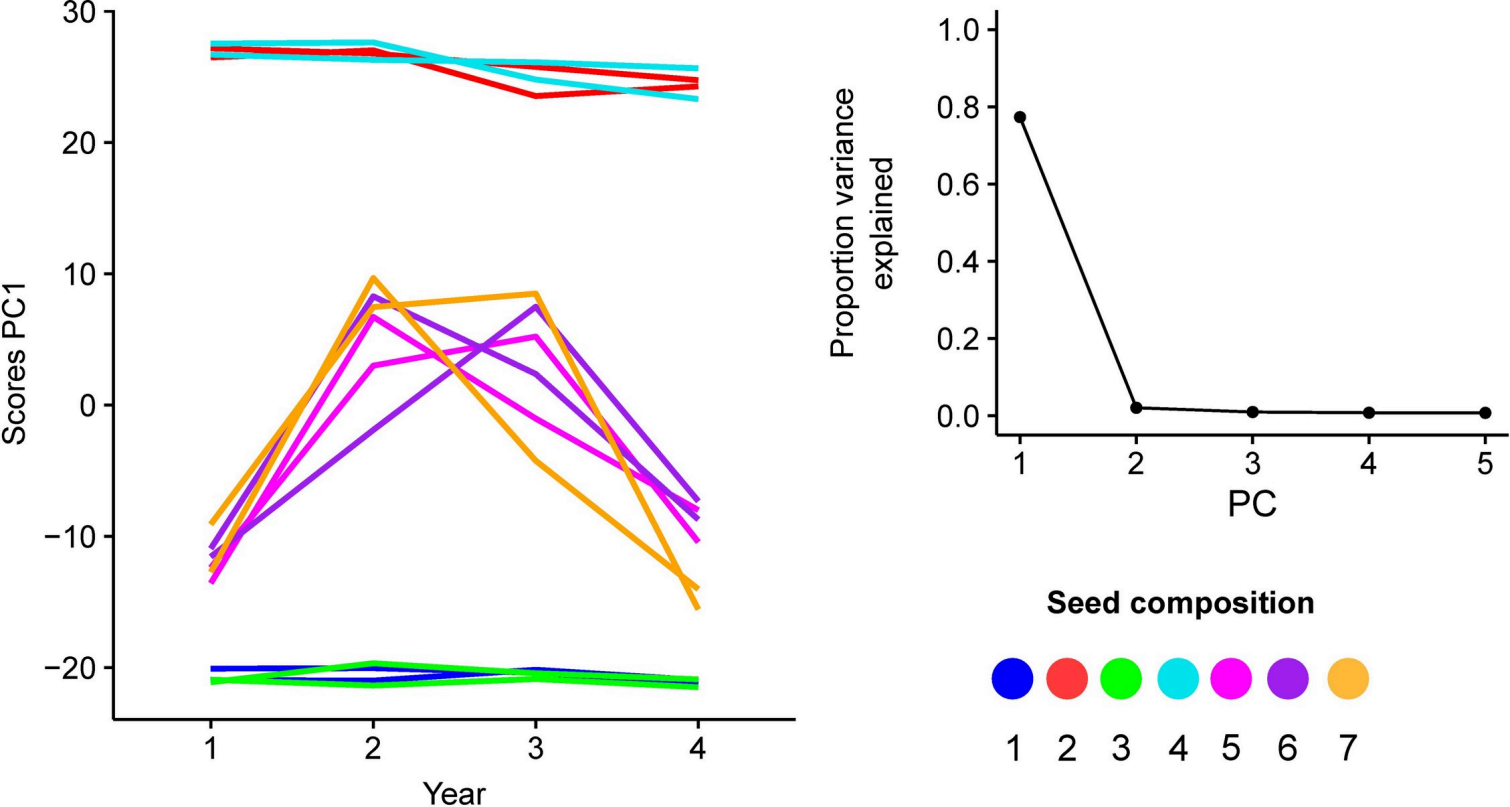
Principal component analysis (PCA). Results for 56 pooled samples based on AAF_pool_ of 22,324 SNPs are summarized. PCA scores of the first PC for each plot over the four years are shown. Lines represent the time series of a field plot, and the colors indicate the seed composition. The screeplot shows the portion of the total variance explained by the first five PCs. The first PC explains 77% of the total variance.

We estimated the overall patterns of differentiation among samples with PCA based on AAF_pool_ of 22,324 SNPs. One meaningful principal component was obtained, explaining 77% of the variance (Fig 3). Samples corresponding to field plots that contained either Merks or Meloni clustered on opposite sides of this PC (seed compositions 1 and 3 versus 2 and 4). Therefore, this PC captures the variance in AAF_pool_ that can be attributed to differentiation between the ryegrass cultivars Merks and Meloni. Samples corresponding to mixtures of both cultivars take an intermediate position on the PC1 axis, and show a rather consistent time-related pattern, with samples taken in years 1 and 4 clustering closer to Merks, and samples taken in years 2 and 3 clustering closer to Meloni.

### Temporal patterns of change in genetic diversity

**Fig 4:**
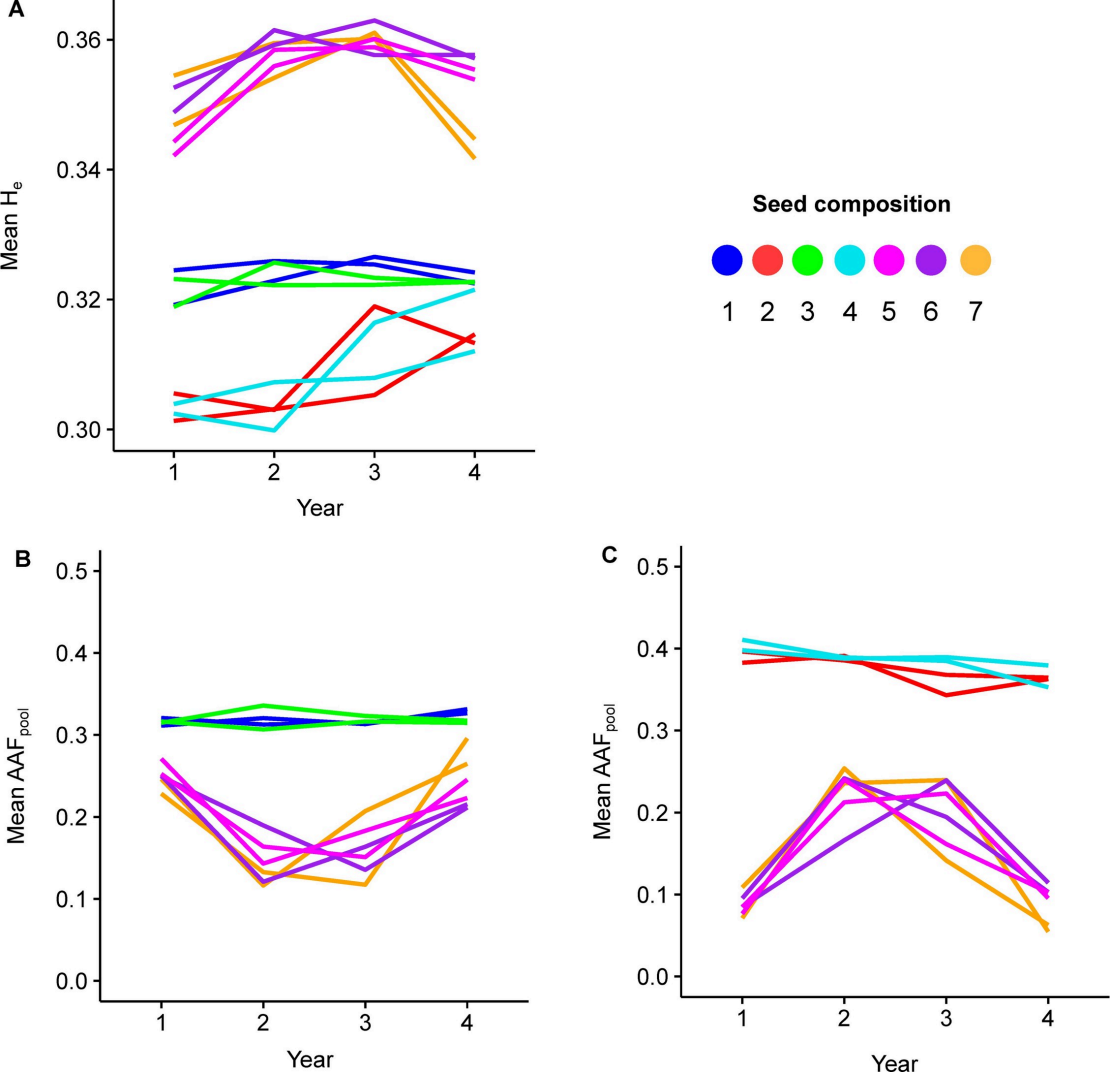
Time series of the genetic diversity of perennial ryegrass swards. Genetic diversity of the 14 field plots as estimated by mean H_e_, based on 22,324 SNPs (**A**), and time series of mean AAF_pool_ per sample, based on 246 Merks private SNPs (**B**) and 617 Meloni private SNPs (**C**). Lines represent the time series of a field plot, and the colors indicate the seed composition. The genetic diversity of field plots with seed compositions containing Merks only (1 and 3) remained stable throughout the four years. The field plots of seed compositions containing Meloni only (2 and 4) were less diverse than those of Merks, but increased slightly after establishment. The field plots containing both cultivars (5 to 7) were more diverse and more variable than the plots containing a single cultivar. These changes coincide with changes in the mean AAF_pool_ of cultivar private SNPs, and are attributed to shifts in the cultivar composition of the population.

The overall genetic diversity of perennial ryegrass populations and its temporal evolution was estimated by calculating mean H_e_ per sample. The main factors explaining mean H_e_ are seed composition S_i_ and its interaction with year (S_i_ x Y_k_), which captures changes in overall genetic diversity that are consistent across replicates (Table 3). The highest mean H_e_ values were found in samples representing plots in which both ryegrass cultivars were sown (seed composition 5, 6 and 7) (Fig 4A). Regarding plots in which only one ryegrass cultivar was sown, samples of Merks (seed compositions 1 and 3) were more diverse than samples of Meloni (seed composition 2 and 4). The genetic diversity of Merks plots remained relatively stable across the four-year experiment, while the diversity of Meloni plots increased. The genetic diversity in plots with both ryegrass cultivars increased from year 1 to year 2, remained constant from year 2 to year 3 and decreased again from year 3 to year 4.

**Table 3:** ANOVA results of the mean H_e_ based on 22,324 SNPs (Fig 4A).

Component	d.f.	SS	MSS	F	P
S_i_	6	2.2 x 10^−2^	3.7 x 10^−3^	293	4.0 x 10^−19^
(S_i_ x R_j_)	7	6.3 x 10^−5^	9.0 x 10^−6^	0.71	0.67
(S_i_ x Y_k_)	21	1.3 x 10^−3^	6.1 x 10^−5^	4.79	3.5 x 10^−4^
ϵ_ijk_	21	2.7 x 10^−4^	1.3 x 10^−5^		

Component is the component of model 4 which were tested, d.f. is the degrees of freedom, SS is the sum of squares, MSS is the mean sum of squares, F is the test statistic and P is the p-value. Both seed composition S_i_ and the interaction of seed composition and year (S_i_ x Y_k_) significantly affect mean H_e_.

The distinct temporal patterns observed in the scores of PC1 (Fig 3) and in the mean H_e_ values (Fig 4A) for the plots in which both ryegrass cultivars were sown suggest that the genetic composition of the mixed ryegrass cultivar plots changed throughout the four years. These changes in the cultivar composition were confirmed by inspection of cultivar private SNPs. In total, 246 (1.1%) and 617 (2.8%) of the 22,324 SNPs were uniquely detected in either Merks or Meloni respectively (Fig 4B and 4C). In both cases, mean AAF_pool_ per sample was relatively constant for ryegrass monoculture plots but displayed temporal changes for the mixed cultivar plots. These results confirm that the cultivar composition of mixed cultivar plots changed over the course of four years. Merks was more prominent in years 1 and 4, Meloni was more prominent in years 2 and 3.

### Variance decomposition of AAF_pool_ frequency data and identification of outlier loci

**Fig 5:**
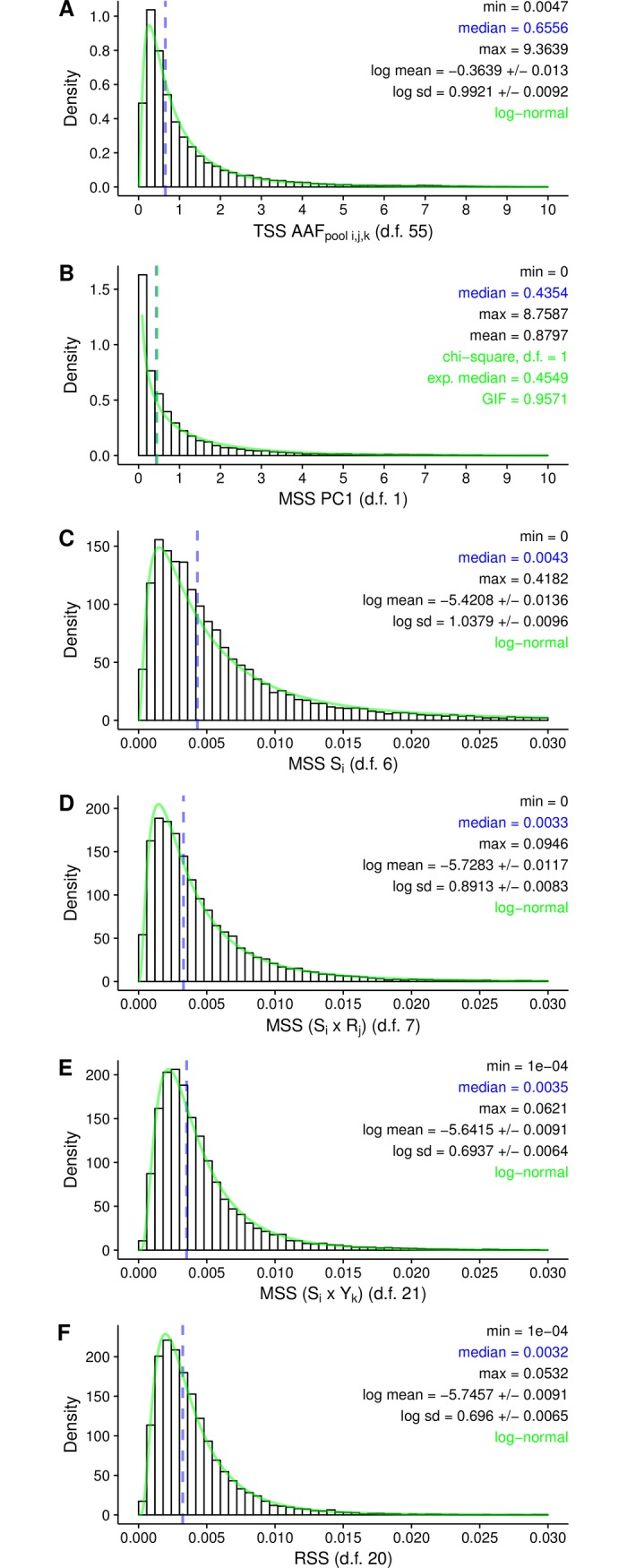
Linear regression of AAF_pool_ based on 22,324 SNPs. Density distributions of the total variance (TSS) **(A)** and the (scaled) variances (MSS) captured by each component **(B-F)** of model 5 (see Material and Methods) are shown. The TSS, the MSS of seed composition S_i_, its interactions with R_j_ and Y_k_, and the RSS follow lognormal distributions. MSS of component PC1 follows a chi-square distribution with one degree of freedom. The green lines represent the predicted probability density functions. The blue dotted lines represent the observed median. The green line represents the expected median of the chi-square distribution.

**Fig 6:**
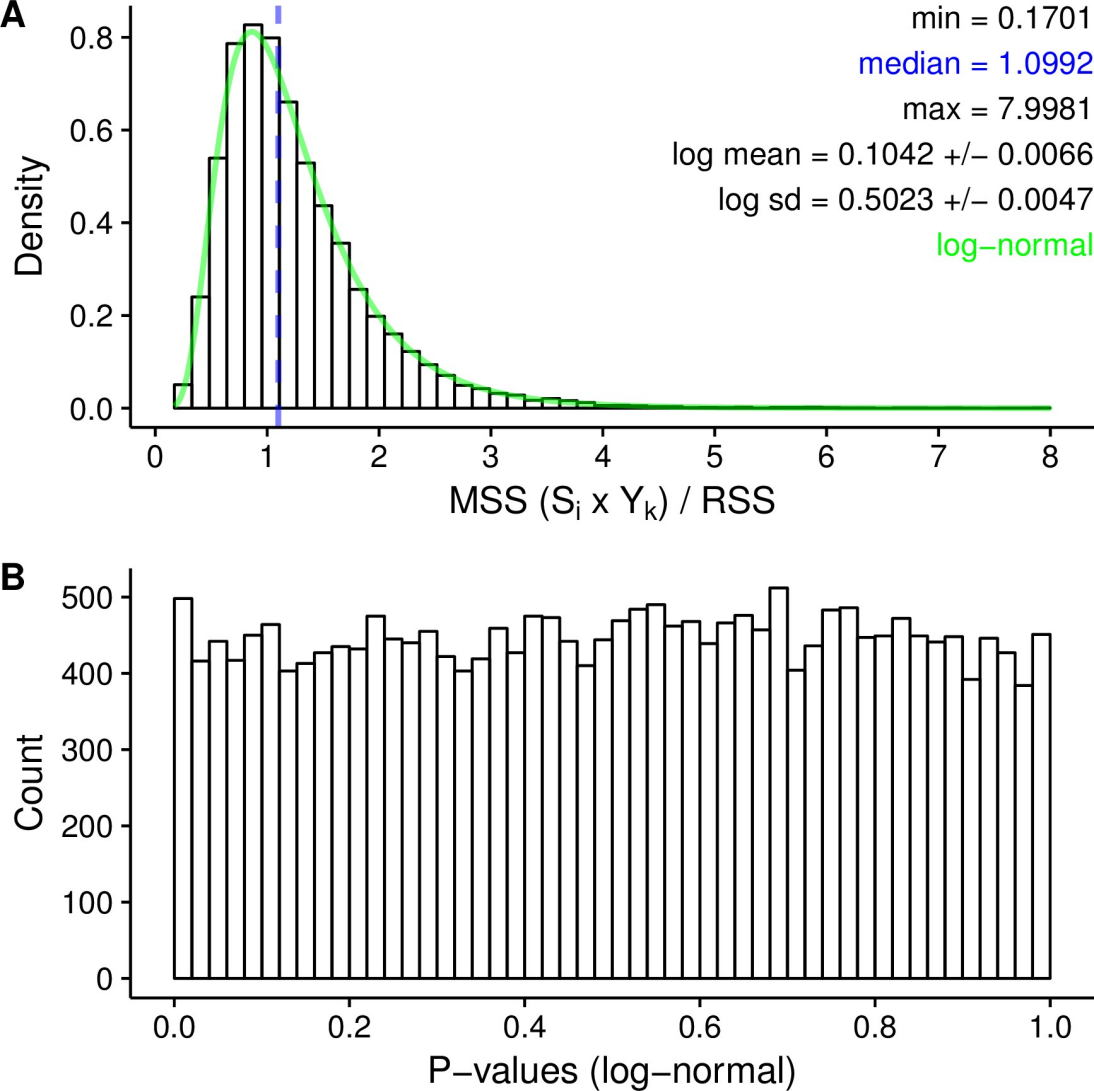
SNP outlier analysis. SNP outlier analysis based on AAF_pool_ to identify loci putatively under selection. Density distribution of the test statistic, i.e. the quotient of MSS of (S_i_ x Y_k_) and RSS (**A**), and distribution of the corresponding p-values (**B**). The blue dotted line indicates the observed median. The green line shows the probability density function of the expected lognormal distribution based on the mean and standard deviation of the test statistic.

The density distributions of the total variance (TSS), the variance captured by each predictor component (MSS) and the residual variance (RSS) are shown in Fig 5. The largest part of the variance was explained by PC1 (median MSS PC1 = 0.4354), which agrees with the results of the PCA (Fig 3, screeplot). Variance due to overall differences among seed compositions (S_i_), to differences between replications (S_i_ x R_j_), or due to temporal changes in AAF_pool_ (S_i_ x Y_k_), were relatively small compared to the residual variance (median MSS of S_i_ = 0.0043; median MSS of (S_i_ x R_j_) = 0.0033; median MSS of (S_i_ x Y_k_) = 0.0035; median RSS = 0.0032). The quotient of MSS of (S_i_ x Y_k_) to the RSS follows a log-normal distribution (Fig 6A). The p-values estimated with the log-normal probability density function based on the observed mean and standard deviation is uniformly distributed (Fig 6B). Therefore, no outlier SNPs or loci putatively under selection were identified.

## Discussion

### Effect of the seed composition on establishment, herbage yield and species abundance

The herbage yield differed among seed compositions throughout the four years of cultivation, including the first year of establishment. This suggests that plant-plant interactions affected biomass production from the establishment of the field and onwards. Fluctuations in yield and species abundance were consistent between field plot replicates. The total DMW peaked during the second year, and competition might have been the most intense during this period.

Red clover often lacks persistence, but in this field trial, the proportion of clover to grass did not decrease, confirming the viability of red clover as a companion for perennial ryegrass [47, 48]. As expected, the presence of clover had a significant positive effect on the total DMW across field plots in which it was combined with perennial ryegrass, despite the fact that plots in which red clover was combined with perennial ryegrass received less N-fertilizer than those in which only ryegrass was sown. Synergy between grasses and legumes is commonly attributed to the nitrogen-fixing capacity of the companion legume [11, 12]. However, other types of species interactions drive grassland dynamics. First, structural complementarity between neighboring ryegrass and clover plants can improve the sward canopy structure (i.e. structural niche differentiation between clover and grass). Secondly, differences in relative growth rates of the component species can affect sward dynamics [49], and biomass production can benefit from asynchrony in growth during the season [50, 51].

It is expected that these functional interactions are affected by the intraspecific diversity of the component species [4]. In this experiment, the intraspecific diversity of the clover component significantly affected total DMW in field plots were both perennial ryegrass cultivars were sown. The erect growing habit of Lemmon is better suited for cultivation under mowing conditions [25], and resulted in a higher clover DMW. In contrast, the ryegrass DMW was the highest in combination with Crossway. This indicates that the functional traits of the clover cultivar affected the competitive interactions between both species. The functional diversity of the ryegrass component also affected herbage yield, and the ryegrass component significantly affected total DMW of field plots were a single perennial ryegrass cultivar was sown. The total DMW was consistently higher for Meloni compared to Merks, both in monoculture and in combination with clover. The interaction of this effect and the effect of the presence of clover was not significant, suggesting that the functional diversity of the ryegrass component did not affect the beneficial interaction between both species. Considering the field plots where two red clover cultivars were sown in combination with one or two perennial ryegrass cultivars, the ryegrass component also significantly affected total DMW. Remarkably, the effect of the ryegrass component was significant for the clover DMW and not significant for the grass DMW. Taken together, these results indicate that the functional diversity of perennial ryegrass affects the growth of red clover, with Meloni being better suited for cultivation in combination with red clover. The intraspecific diversity of both species affects functional interactions within grassland swards.

### Validation of the pool-GBS approach

Single nucleotide polymorphisms (SNPs) have proven to be effective to characterize the genetic diversity among plant populations. Using whole genome shotgun sequencing (pool-seq), allele frequencies can be estimated directly from pools of plants [52–54]. Yet this protocol is still costly for sequencing many samples, especially for species with large genomes. Complexity reduction methods such as GBS target a fraction of the genome associated with restriction sites, thereby reducing the sequencing cost per sample [29, 55, 56]. The combination of pool-seq and GBS (pool-GBS) is a promising strategy for assessing genetic diversity among populations on a genome-wide scale [57]. This approach has previously been applied to perennial ryegrass [26, 27], barley [58, 59], multiple species of alpine shrubs [60], herring [61], and cyst nematodes [62]. To the best of our knowledge, this approach has not been used before for the characterization of temporal changes in the genetic composition of plant populations. Therefore, we considered the potential limitations of this methodological approach. The pool-GBS procedure was optimized towards three main criteria: read data completeness, accuracy of measurements of allele frequencies and removal of non-reproducible SNPs.

A central goal of this study was to compare quantitative estimates of genetic diversity across populations. Estimation of the diversity index mean H_e_ strongly depends on the set of genomic loci under consideration. For comparison of samples, mean H_e_ is preferentially calculated for a common set of loci. High proportion of missing data have been reported for GBS data [29, 63]. Therefore, we investigated the read data distribution of the pool-GBS procedure in a validation experiment, and determined a suitable amount of read data per sample to minimize missing data. For the 56 pooled samples, we obtained consistent sampling of ca. 0.1% of the perennial ryegrass reference genome with a very low level of missing data across populations. Creating two independent tissue pools per population and merging the read data of such replicated libraries increased the number of consistently detected loci further.

As AAF_pool_ is directly estimated from the read counts at a certain locus, genotyping errors can be confounded with low frequency alleles [42, 53]. Moreover, the accuracy of the allele frequency measurement is affected by the number of plants per pool [57, 64, 65]. While Byrne [26] and Ashraf [27] sampled several hundreds of perennial ryegrass seedlings to represent the population genetic parameters, our representation of the genetic diversity of a field plot relied on sampling of leaf material from 40 randomly selected individual plants per field plot. Therefore, we empirically validated the accuracy of allele frequencies obtained with pool-GBS. AAF_pool_ estimated in three replicate pools correlated very well with the AAF_ind_ estimated from individual GBS genotyping. However, pool-GBS was sensitive to genotyping errors, and stringent filtering of the SNP loci was required to identify a reliable set of 22,324 high quality SNPs across the 56 population samples.

### Genetic characterization and temporal dynamics of the ryegrass component

The perennial ryegrass cultivars could be differentiated with PCA based on the AAF_pool_ of 22,324 SNPs_,_ similarly as in Byrne [26]. The genetic differentiation between Merks and Meloni represented the majority of the variance in the AAF_pool_ dataset. perennial ryegrass cultivars are genetically very diverse [16–18], and some genotypes within a sward might be better adapted to the prevailing conditions. Moreover, these conditions change during the cultivation period, e.g. changes in the biomass production and competitive pressure within the sward, and variability of the environmental conditions. Therefore, we expected changes in the genetic composition of the ryegrass component. The largest changes in the genetic composition of the ryegrass populations were observed in field plots containing both ryegrass cultivars. The representation of the cultivars in the plots where they are sown together changed throughout the cultivation period. This illustrates the dynamic behavior of the ryegrass component in mixed cultivar field plots. If a cultivar is less prominent at a certain point in time, it is not necessarily removed from the population. Changes in cultivar abundance might be related to their functional characteristics, similar to changes in species abundance [66]. Similar dynamics have been described between grass species by Brophy [49]. They observed that species dynamics are primarily driven by relative growth rates, and secondarily by density dependent and climatic factors. However, it was concluded that the species with the highest relative growth rates became dominant over time. In this study we did not observe either a clear dominance of one of the species nor one of the cultivars. In field plots containing a single perennial ryegrass cultivar, we did not detect pronounced genetic fluxes. The genetic diversity of Merks populations remained stable across the four years, while the genetic diversity of the Meloni populations showed a slight increase. This may be related to the decrease of grass biomass production towards the third and the fourth year of cultivation. It is possible that a subset of Meloni genotypes that was more dominant during the first and second year, became less dominant in subsequent years, increasing the chance of sampling a more diverse set of genotypes.

### Detection of loci putatively under selection

We developed a statistical test to identify loci that significantly change in time. However, we did not identify convincing signatures of selection in the perennial ryegrass populations investigated. Possibly, selection pressures were too low to have any significant detectable effect on AAF_pool_ data. An alternative explanation for not detecting outlier loci is related to linkage disequilibrium (LD) patterns in these populations. With the current spacing of GBS tags across the genome (approx. 1 stack of 100 bp per 100-200kbp), the genetic markers observed may not be in genetic linkage with loci under selection, if LD extends only for short distances [67]. In this situation, genome complexity reduction approaches such as GBS are likely to miss the majority of outliers that might be present [68].

## Conclusions

The interactions between neighboring plants plays an important role in the functioning of cultivated grasslands, and are determined by the functional diversity of the sown species and cultivars. This study showed that herbage yield and species abundance of field plots is affected by the mixture of perennial ryegrass and red clover cultivars used. Genetic diversity among ryegrass populations, investigated by GBS genotyping of pooled samples, showed that the abundance of perennial ryegrass cultivars is highly dynamic in mixtures. Taken together, these results illustrate that phenotypic traits of both perennial ryegrass and red clover affect their behavior in seed mixtures of both species and that the dominance of cultivars in mixtures can shift throughout the cultivation period.

## Supporting information

S1 Table Fertilization of the field plots. (XLSX)

S2 Table Yield data. (TSV)

S1 Fig Saturation curves. Saturation curves show the relationship between the number of reads per sample (x-axis) and the number of base positions of the perennial ryegrass reference genome (Byrne *et al*., 2015) that are covered (y-axis) at various minimum RD threshold; RD 10 (blue), RD 30 (red), RD 100 (orange) and RD 300 (green). **A** shows the data of individual plants (between 0 and 4 M reads), data of three replicate pools (between 12 and 14 M reads) and pairwise merged data of pools (between 26 and 30 M reads) of the validation experiment. The line curves were constructed by resampling reads of the validation experiment. These curves suggest that the larger part of potentially available GBS loci are covered if at least ~20 M reads are obtained per sample. **B** shows the data for the 56 population samples of the field experiment. Data of the technical replicates (between 0 and 14 M reads), were merged for each population sample (between 20 and 54 M reads) (see pooling and replication scheme Fig 1).
(PDF)

S2 Fig Allele frequency correlations. Allele frequency correlations of SNPs that were identified in a set of 40 plants (AAF_ind_) and three pool replicates of the same set (AAF_pool_), showing the number of SNPs (n), the median deviation (d), Pearson’s correlation coefficient (r) and the least squares regression (red). **A** The effect of SNP filtering on the correlation of AAF_ind_ and AAF_pool_. The SNPs of the individuals were filtered on maximum missing data (MD) 1, 5 or 10 out of 40 samples, and the SNPs of the pool were filtered on minimum RD of 30, 100 or 300. For subsequent comparisons we consistently the thresholds RD 30 and MD 5 **B:** Correlation of AAF_pool_ of the three pool replicates to AAF_ind_. **C:** Pairwise correlations of AAF_pool_ of the three pool replicates. Only SNPs that were also detected in the individuals were considered for this comparison. **D:** Correlation of AAF_pool_ obtained by pairwise merging of pool replicates to AAF_ind_.
(PDF)

S3 Fig Venn diagrams of overlapping SNPs. Venn diagrams showing the number of SNPs that were identified in individual samples, pooled samples, or both. The comparisons of SNP datasets of **A—D** follows the same order as the allele frequency correlations (S2 Fig). **E** Venn diagram of heterozygous loci across three replicates of one individual plant, and three-way comparison of the three pool replicates.
(PDF)

S4 Fig Pairwise comparisons of replicate pools. Pairwise comparisons of AAF_pool_ distributions of replicate pools. Each distribution shows the AAF_pool_ distribution of a pooled sample, colors indicate whether the SNP was detected in the corresponding replicate (blue for detected, red for not detected and black for no data available). Non-reproducible SNPs are strongly skewed towards low AAF_pool_ values.
(PDF)

## Acronyms

AAF_ind_: alternative allele frequency estimated from individual sample
AAF_pool_: alternative allele frequency estimated from pooled sample
DMW: dry matter weight
GBS: genotyping-by-sequencing
LD: linkage disequilibrium
MAC: minor allele read count
MSS: mean sum of squares
PCA: principal component analysis
PCR: polymerase chain reaction
pool-GBS: genotyping-by-sequencing of pooled samples
pLRT: p-value of likelihood ratio test
RSS: residual sum of squares
SNP: single nucleotide polymorphism
TSS: total sum of squares
