# Supplementary figures and images for: Temporal changes in genetic diversity and forage yield of perennial ryegrass in monoculture and in combination with red clover in swards
Source: PLoS One. 2018 Nov 8;13(11):e0206571. doi: 10.1371/journal.pone.0206571 (PMC6224058; doi:10.1371/journal.pone.0206571)

**A**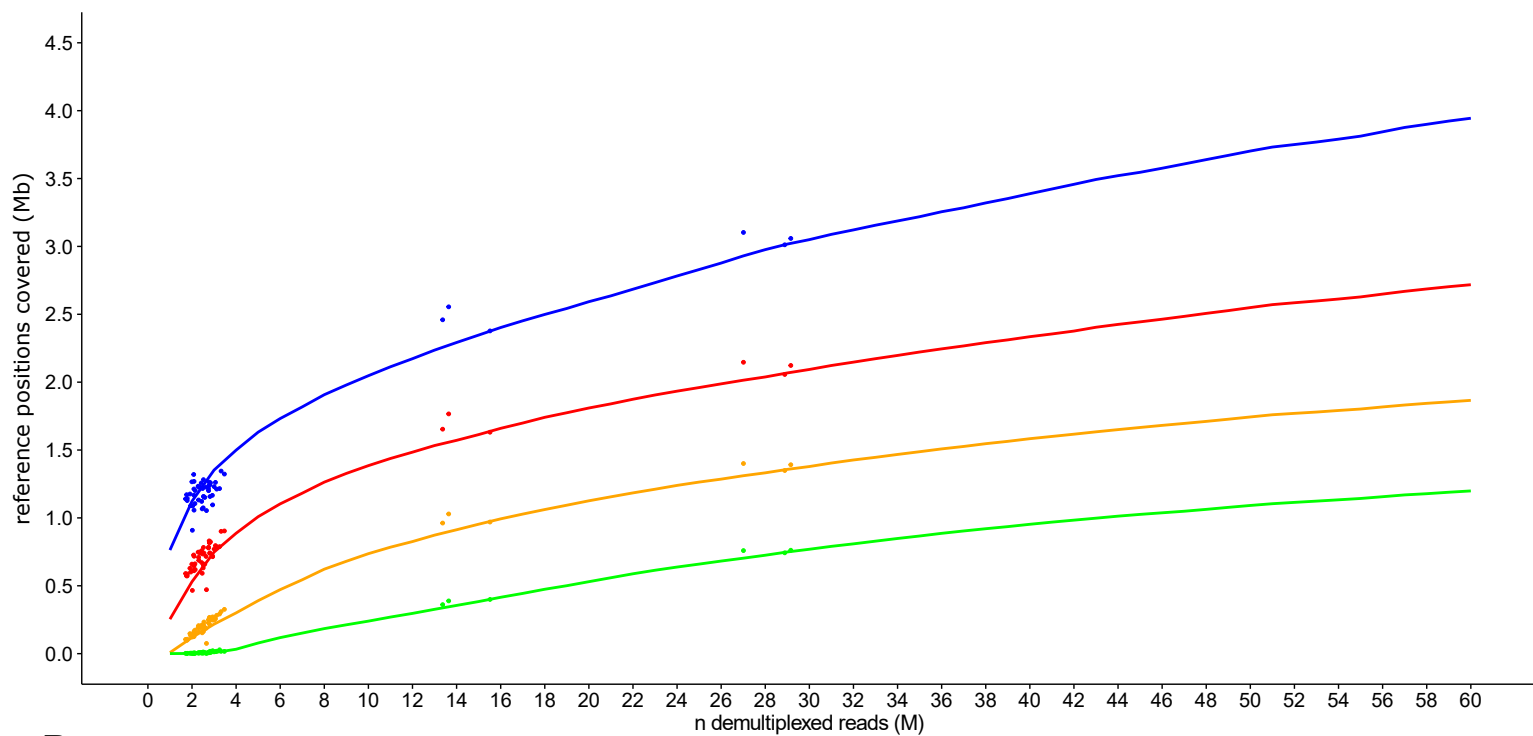**B**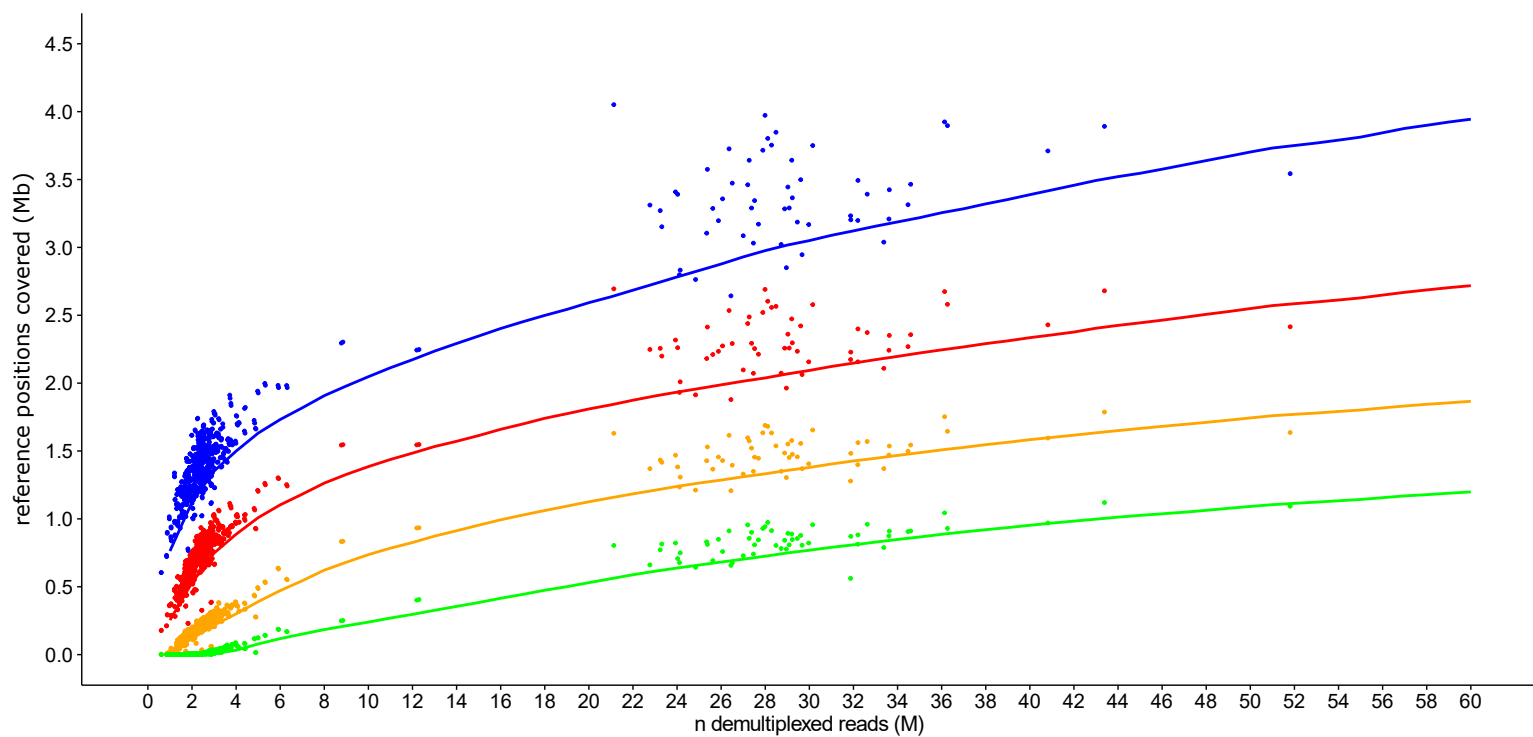

Supplement: S1 Fig — Saturation curves show the relationship between the number of reads per sample (x-axis) and the number of base positions of the perennial ryegrass reference genome (Byrne et al., 2015) that are covered (y-axis) at various minimum RD threshold; RD 10 (blue), RD 30 (red), RD 100 (orange) and RD 300 (green). A shows the data of individual plants (between 0 and 4 M reads), data of three replicate pools (between 12 and 14 M reads) and pairwise merged data of pools (between 26 and 30 M reads) of the validation experiment. The line curves were constructed by resampling reads of the validation experiment. These curves suggest that the larger part of potentially available GBS loci are covered if at least ~20 M reads are obtained per sample. B shows the data for the 56 population samples of the field experiment. Data of the technical replicates (between 0 and 14 M reads), were merged for each population sample (between 20 and 54 M reads) (see pooling and replication scheme Fig 1). (PDF) [file pone.0206571.s003.pdf]

**A****individuals****MD 1**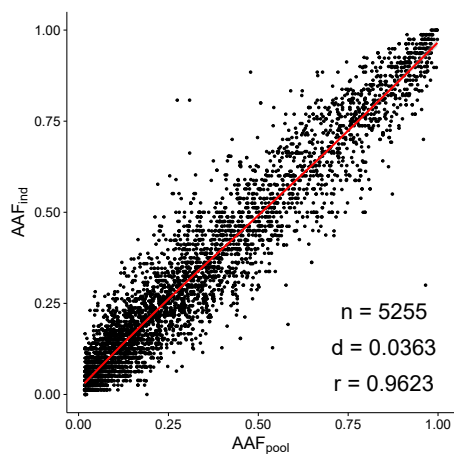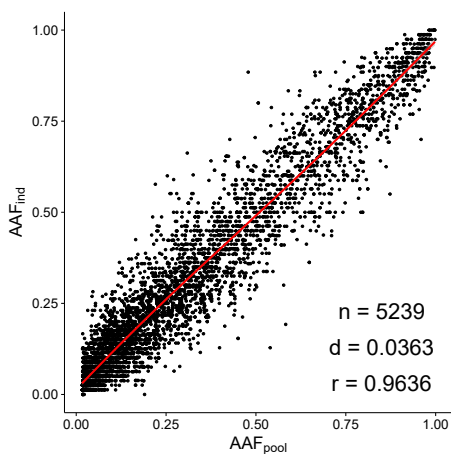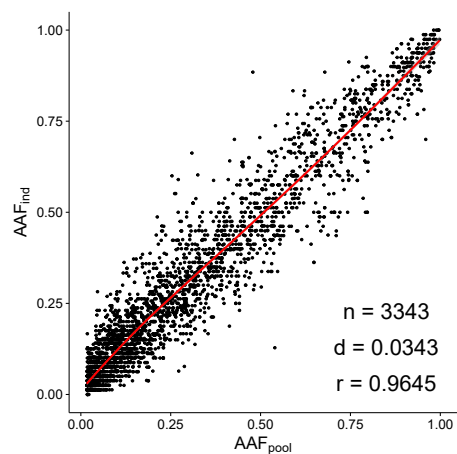**MD 5**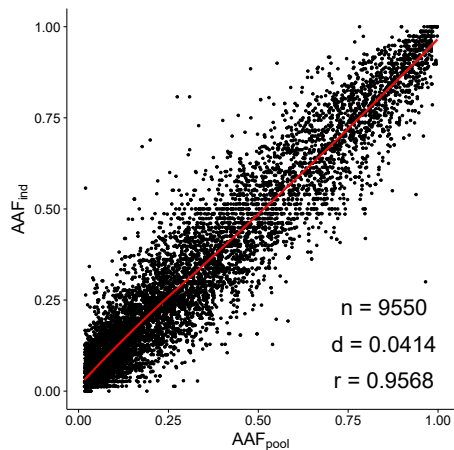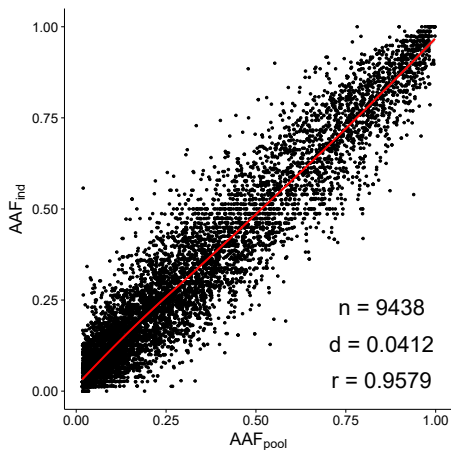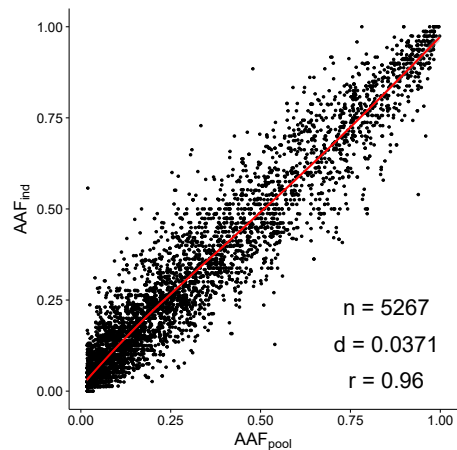**MD 10**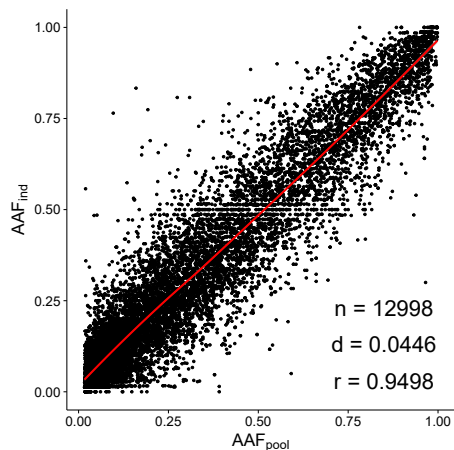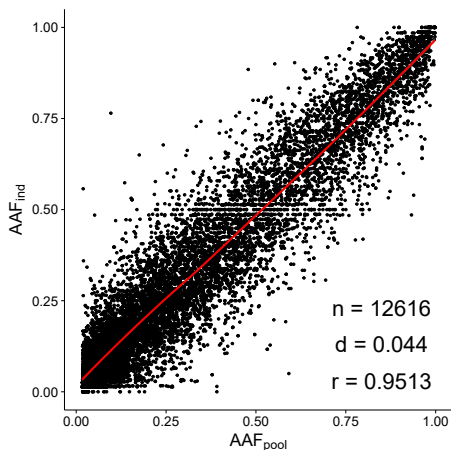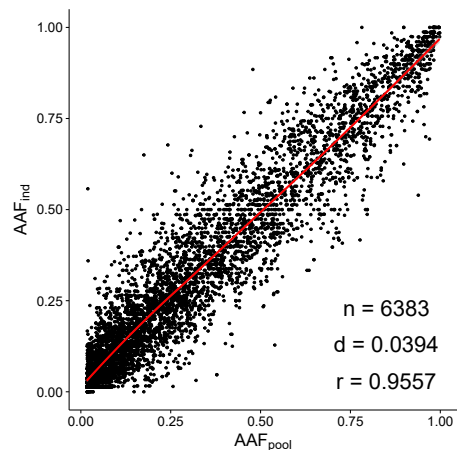**RD 30****RD 100****RD 300****pool**

**B**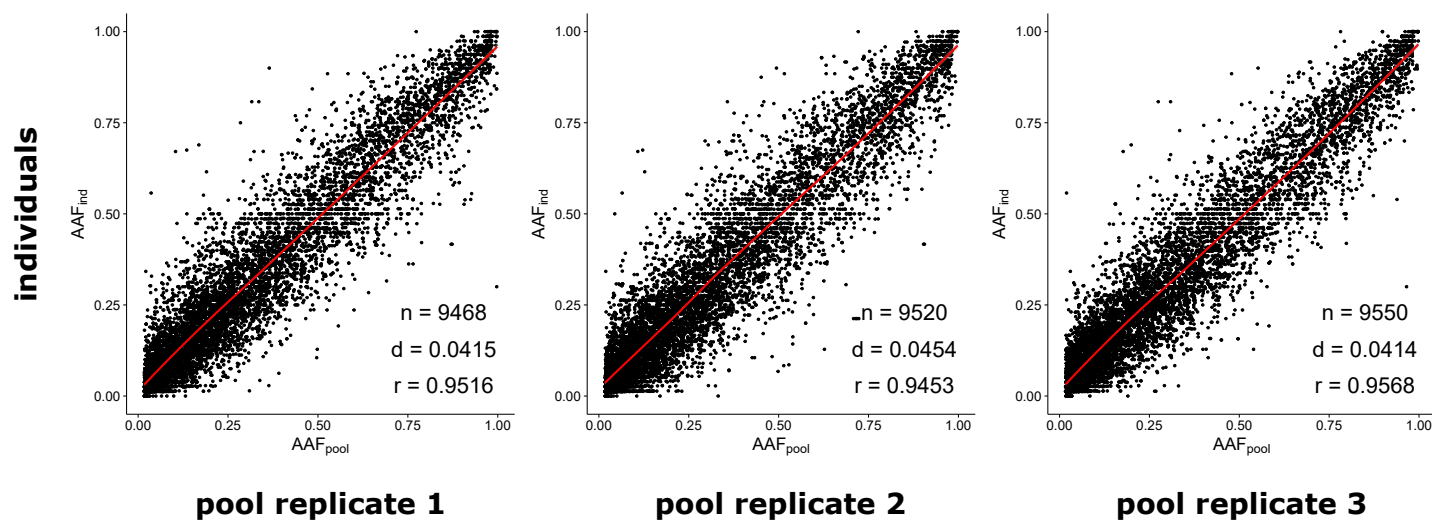**C**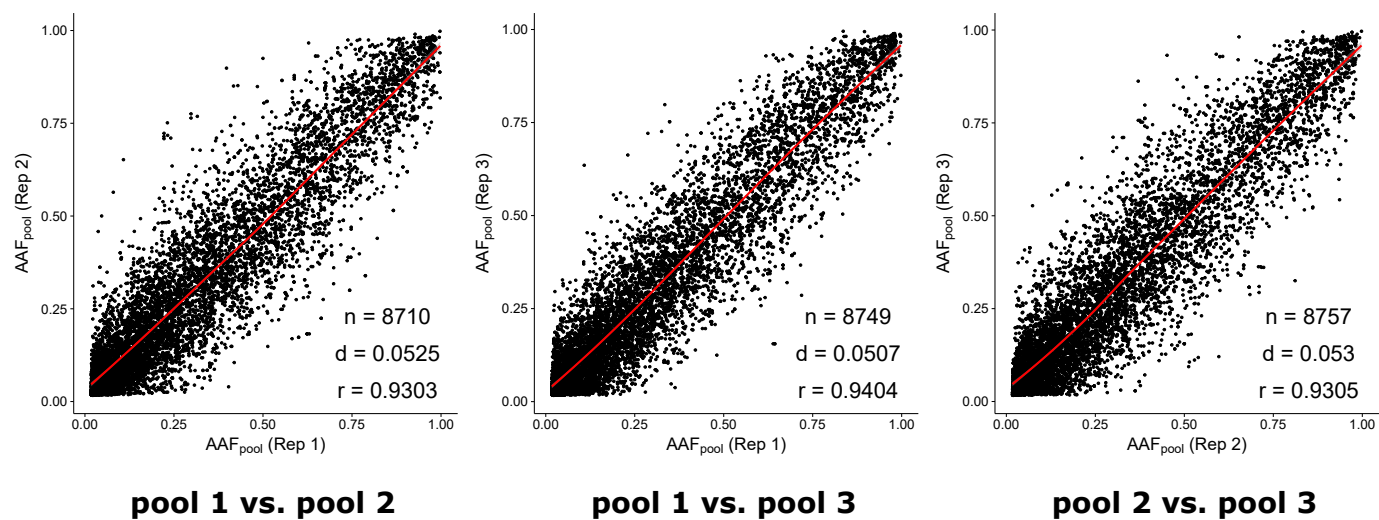**D**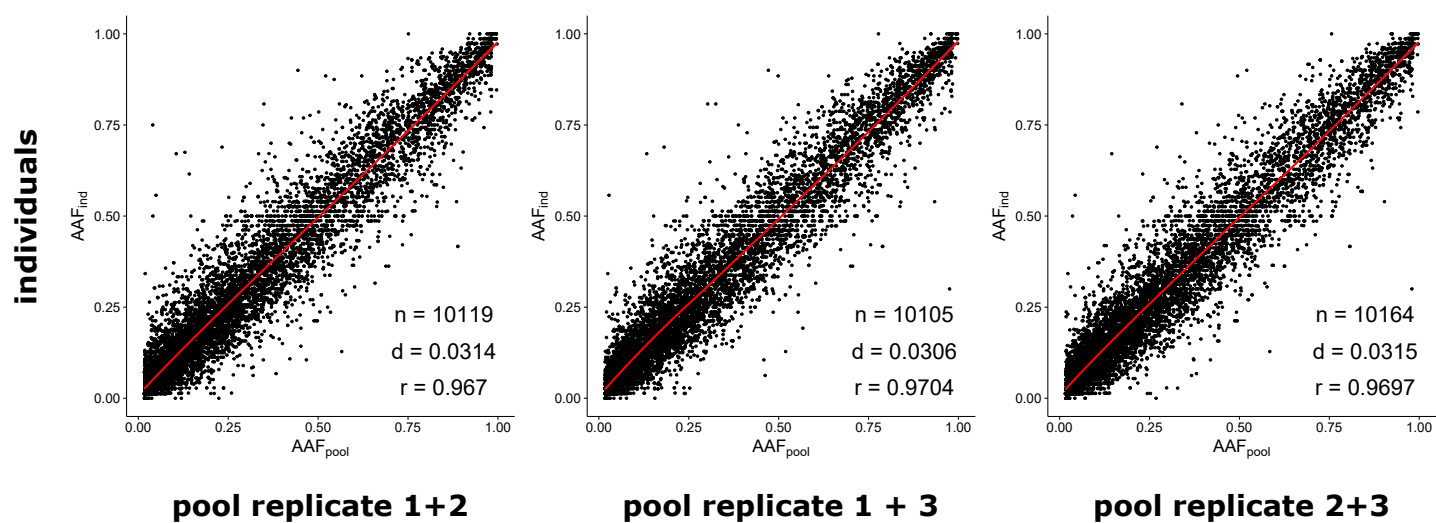

Supplement: S2 Fig — Allele frequency correlations of SNPs that were identified in a set of 40 plants (AAFind) and three pool replicates of the same set (AAFpool), showing the number of SNPs (n), the median deviation (d), Pearson’s correlation coefficient (r) and the least squares regression (red). A The effect of SNP filtering on the correlation of AAFind and AAFpool. The SNPs of the individuals were filtered on maximum missing data (MD) 1, 5 or 10 out of 40 samples, and the SNPs of the pool were filtered on minimum RD of 30, 100 or 300. For subsequent comparisons we consistently the thresholds RD 30 and MD 5 B: Correlation of AAFpool of the three pool replicates to AAFind. C: Pairwise correlations of AAFpool of the three pool replicates. Only SNPs that were also detected in the individuals were considered for this comparison. D: Correlation of AAFpool obtained by pairwise merging of pool replicates to AAFind. (PDF) [file pone.0206571.s004.pdf]

**A**

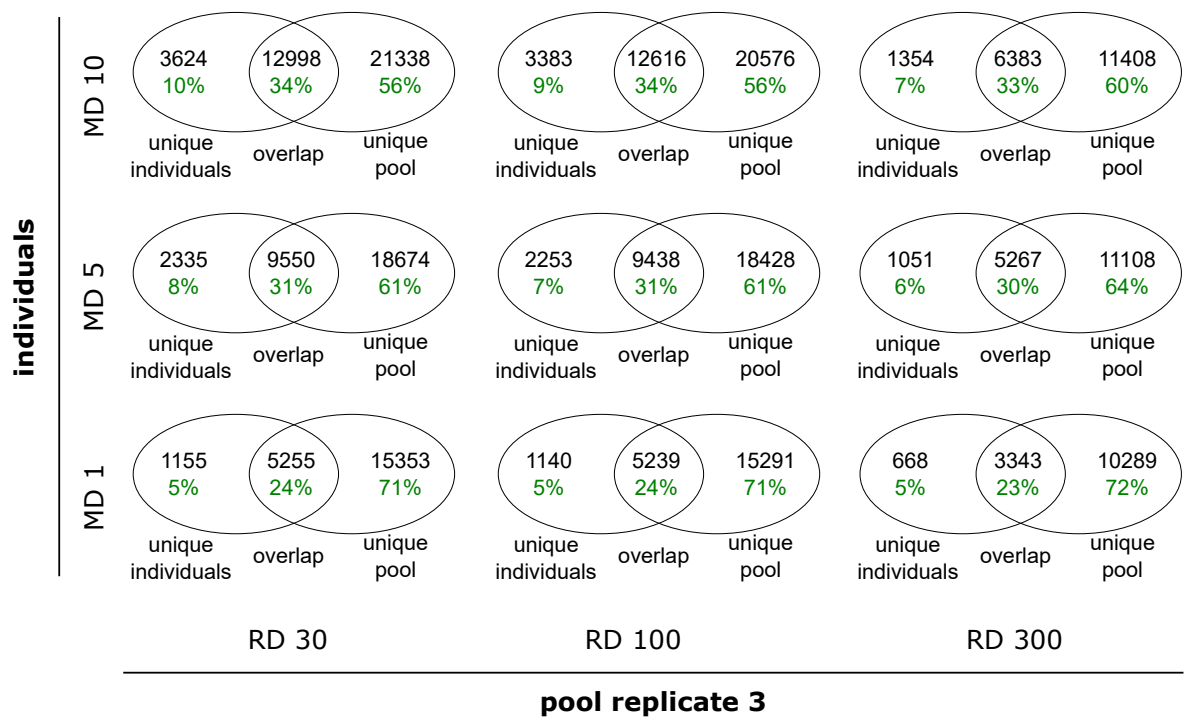

**B**

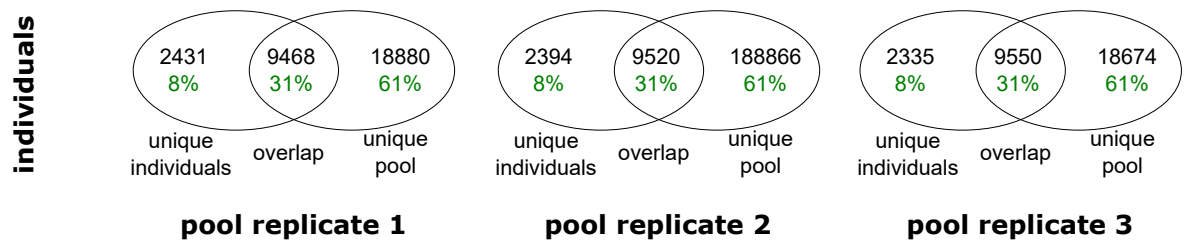

**C**

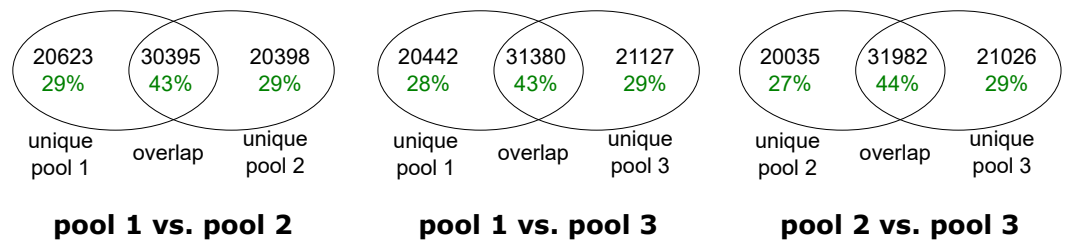

**D**

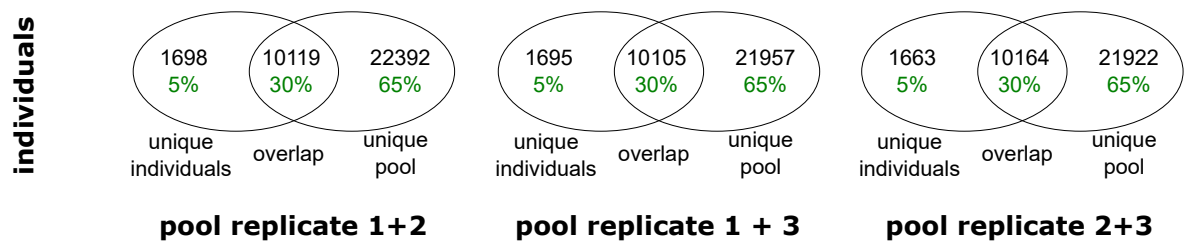

**E**

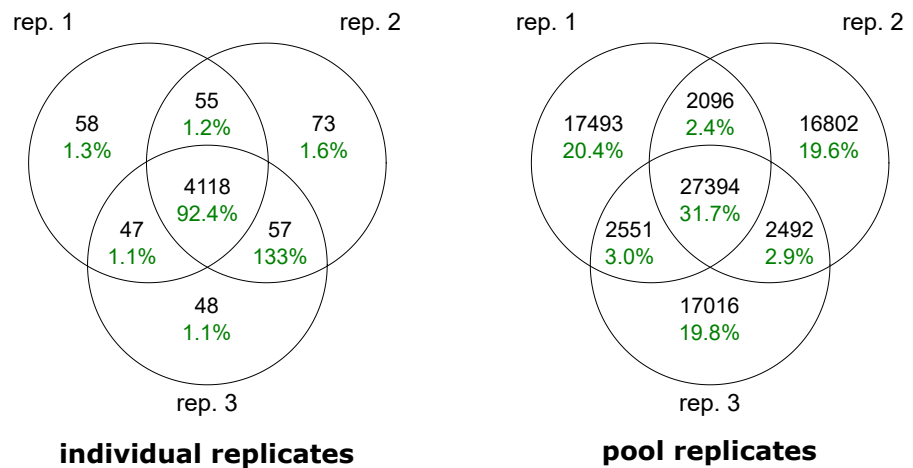

Supplement: S3 Fig — Venn diagrams showing the number of SNPs that were identified in individual samples, pooled samples, or both. The comparisons of SNP datasets of A—D follows the same order as the allele frequency correlations (S2 Fig). E Venn diagram of heterozygous loci across three replicates of one individual plant, and three-way comparison of the three pool replicates. (PDF) [file pone.0206571.s005.pdf]

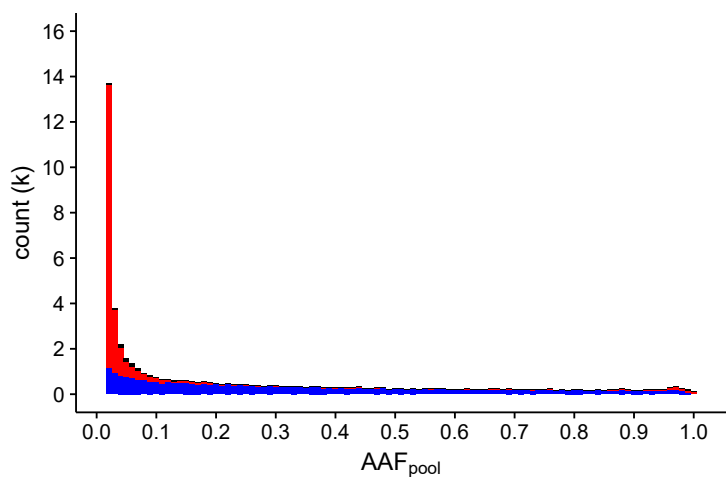

**pool 1 vs. pool 2**

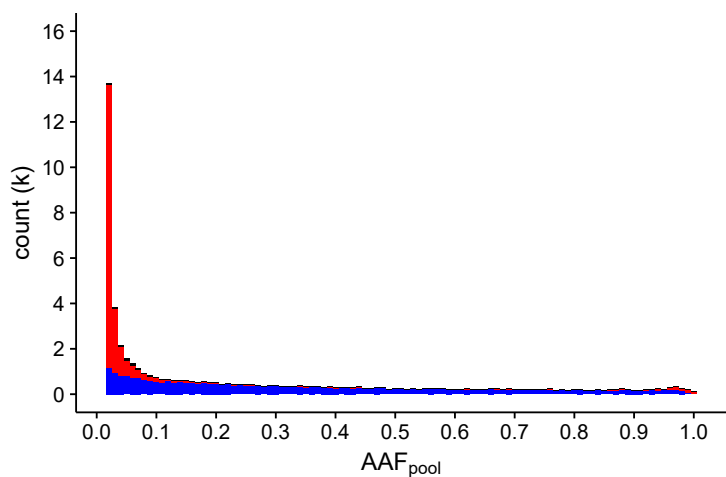

**pool 1 vs. pool 3**

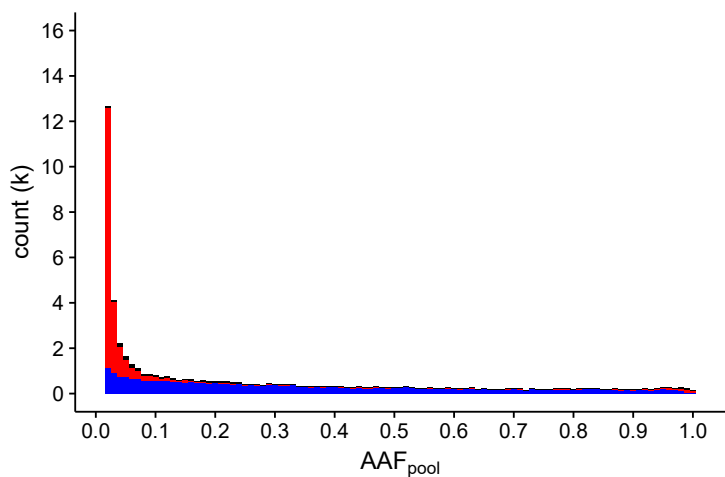

**pool 2 vs. pool 1**

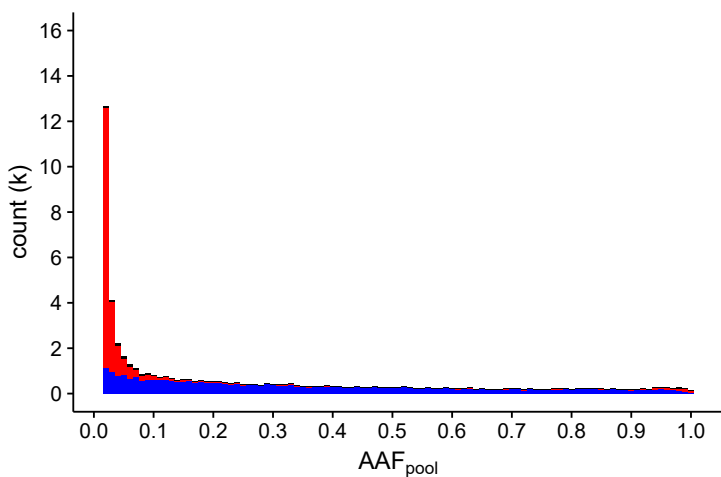

**pool 2 vs. pool 3**

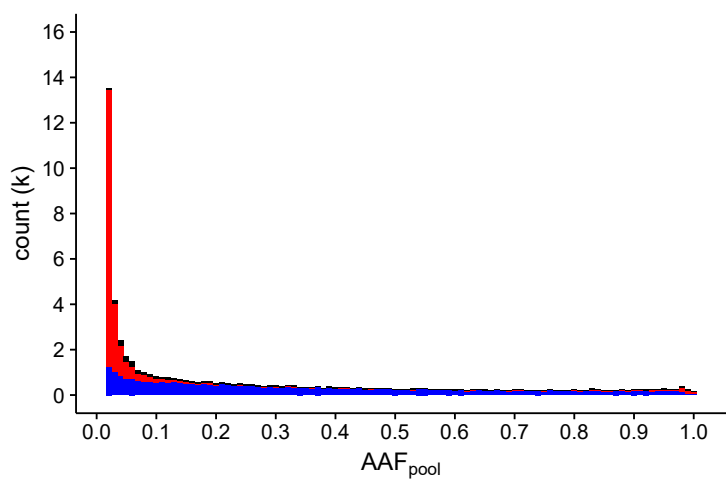

**pool 3 vs. pool 1**

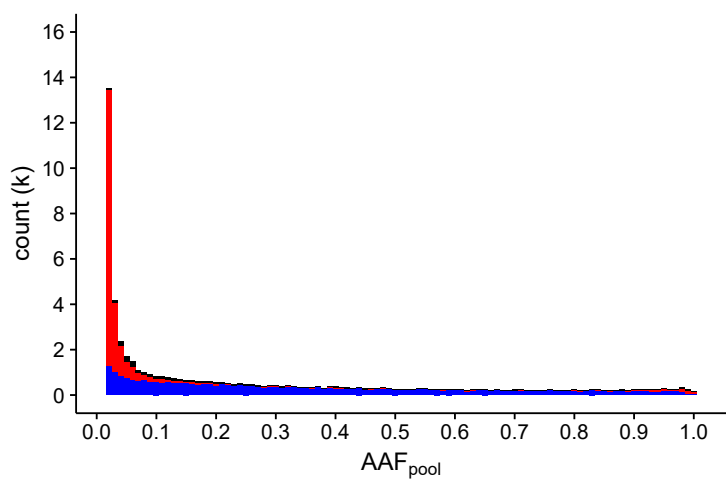

**pool 3 vs. pool 2**

Supplement: S4 Fig — Pairwise comparisons of AAFpool distributions of replicate pools. Each distribution shows the AAFpool distribution of a pooled sample, colors indicate whether the SNP was detected in the corresponding replicate (blue for detected, red for not detected and black for no data available). Non-reproducible SNPs are strongly skewed towards low AAFpool values. (PDF) [file pone.0206571.s006.pdf]
